# The intricate diversity of human–nature relations: Evidence from Finland

**DOI:** 10.1007/s13280-023-01933-1

**Published:** 2023-09-29

**Authors:** Kaisa J. Raatikainen, Anna-Kaisa Tupala, Riikka Niemelä, Anna-Mari Laulumaa

**Affiliations:** 1 Societal Change Unit, Finnish Environment Institute (Syke), Survontie 9A, 40500 Jyväskylä, Finland; 2 Department of Biological and Environmental Science, School of Resource Wisdom, University of Jyvaskyla, P.O.Box 35, 40014 Jyväskylä, Finland; 3 Regional Council of Central Finland, Lutakonaukio 7, 40100 Jyväskylä, Finland; 4 School of History, Culture and Art Studies, University of Turku, 20014 Turku, Finland; 5 Kiertotie 12 as 2, 40250 Jyväskylä, Finland

**Keywords:** Art&Science, Mixed-methods, Nature connectedness, Relationalism, Sustainability, Transdisciplinary research

## Abstract

Supporting sustainability requires understanding human–nature relations, which we approached as social constructions that can be studied through nature-related discourses. We examined human–nature relations in Finland by combining approaches from environmental social sciences and arts-based research into a mixed-methods design. A public online survey (*n* = 726) and post-performance audience interviews (*n* = 71) portrayed nature positively. Respondents’ ideas of nature ranged from natural scientific to philosophical; from dualistic to holistic; and from ecocentric to anthropocentric. A factor analysis revealed discourses focusing on wellbeing, conservation, ecoanxiety, pro-environmentalism, outdoor activity, and enjoying nature. Interviews added spiritual and over-generational aspects and revealed the importance of embodied experiences in nature relations. We identified dimensions that structure the relations, including human–nature positionality, engagement and contact with nature, and conception and thought. The emotional and experiential aspects, and nature-related practices, deserve further research. We demonstrate how a diversity of human–nature relations co-exists and co-evolves.

**Supplementary Information:**

The online version contains supplementary material available at 10.1007/s13280-023-01933-1.

## Introduction

People are connected to nature in various ways, which range from material dependencies to emotional and philosophical linkages (Ives et al. 2018). In the twenty-first century, these society–nature relations have gained unforeseen breadth, depth, and consequentiality—but in a worrying manner (Castree 2001). Modern societies are claimed to be distanced from nature, contributing to the ongoing ecological crisis and hindering efforts to solve environmental issues (e.g., Ives et al. 2017, 2018; IPBES 2019, 2022; Barragan-Jason et al. 2022). A recent global meta-analysis (Barragan-Jason et al. 2022) demonstrated that the level of human–nature connectedness, i.e., the extent to which humans consider themselves as part of nature, corresponds to sustainability-oriented and pro-environmental mindsets and behaviors. Thus, it is logical to suggest that strengthening the connections between people and nature and uncovering the emotional attachment and positive values tied to nature support sustainability (Lumber et al. 2017; Ives et al. 2018; Yletyinen et al. 2022). People value nature differently across a range of worldviews and knowledge systems. Recognizing and respecting this plurality would benefit both people and nature (IPBES 2022).

Environmental education is the most popular means to cultivate pro-environmentalism, but it has proven inefficient in increasing pro-environmental behavior and individuals’ perceived connectedness to nature (Lumber et al. 2017; Barragan-Jason et al. 2022). This finding emphasizes the need to understand the complexity and resilience of cognitive frameworks on which people base their actions in respect to nature. Supporting environmentally beneficial human–nature relations is difficult in industrialized countries that predominantly prioritize instrumental values of nature (IPBES 2022). Research can help solving this challenge by unveiling the complex ways in which people relate to nature, discovering how to strengthen nature connectedness, and seeking ways to bring people closer to nature. For example, there is evidence that guided walks in nature increase the sense of nature connectedness, if the walk is enriched with sensory and emotional activities (Lumber et al. 2017). Also, arts-based practices increase environmental sensitivity and engagement with nature through inclusion of hands-on activities and emotional aspects (Raatikainen et al. 2020).

In this paper, we empirically examine the diversity of human–nature relations in Finland through a relationalist lens. With relationalism we refer to the idea that social phenomena are produced through dynamic interactions (Dépelteau 2018). Human–nature relations are, by default, based on interactions between people and nature. Human–nature relations are complex and everchanging (e.g., Williams 1980) and thereby appear elusive. Our research set out to study the diverse ways in which people and nature connect to each other and the multiple dimensions wherein human–nature relations evolve. We adopted a transdisciplinary research methodology based on art&science collaboration. Arts-based research methodologies are increasing in popularity in social sciences as they reach non-verbal, embodied and experiential types of knowledge, which are unattainable through more traditional research methods (Coutts et al. 2018; Chilton and Leavy 2020). Mixing qualitative and quantitative methods from the social sciences with post-performance audience interviews allowed us to gain a more comprehensive understanding on human–nature relations.

Our analytical starting point was to approach”nature” both as a physical actor and a concept that evolves in the interaction between humans and nature. Nature refers to physical things, but is also a social construction that allows people to position themselves in the world (Williams 1980; Castree 2001). Physical nature is an interactant, i.e., a thing that acts and is acted upon. Therefore, human–nature relations are reciprocal and processual: people’s actions have an impact on physical nature and simultaneously engagement with nature affects people (Schroeder 2007).

People situate themselves with respect to nature through discourses that define nature. Studying nature-related discourses enables grasping the reasoning behind different views of nature, understanding how attitudes and behaviors towards nature are formed (Muradian and Pascual 2018), and investigating how discourses enable related types of actions (Hugé et al. 2013). Based on these premises, we studied human–nature relations through data derived from a public survey and audience interviews. By applying arts-based inquiry we extended the analysis to the embodied and emotional aspects of experiencing nature. This mixed-methods approach allowed us to clarify how people understand, define, and act on nature (Hugé et al. 2013); and to shed light on the diversity of values shaping human–nature relations (Muradian and Pascual 2018).

We conducted our research in Finland, addressing the Finnish-speaking population. Finland is a North European welfare state, with ca. 70% of the gross domestic product derived from the services sector and 72% of the population urban (Statistics Finland 2023). Forests cover 75% of the area of Finland (Ministry of Agriculture and Forestry 2023), and the use of forests has been an important driver for economic development (Björn 2000) and biodiversity loss (Kontula and Raunio 2018). Finns have access to nature based on the rights to roam (i.e., the legitimate right to responsibly move, camp, and forage in nature regardless of landownership). The importance of natural resource use and unrestricted access to nature are often mentioned as contributing to the appreciation of nature, specifically forests, in the Finnish society (Sitra and Kantar TNS 2021; Björklund et al. 2022; Finnish Environment Institute 2022). However, this involves contradictions between utilitarian and intrinsic values (Björklund et al. 2022). As such, Finland represents a case as a country in which industrialized market economy co-exists with a wide-spread cultural appreciation of nature.

From these points of departure, we formulated three research questions:

- How nature is conceptualized in Finland in terms of associative words and freely formulated definitions? (RQ1)
- Which discourses inform the ways in which people relate to nature in Finland, and which generalizable dimensions of human–nature relations can be found in these discourses? (RQ2)
- What kinds of embodied and emotional nature experiences emerged from participating in a site-specific walking performance? (RQ3)

The next section explains how and why we applied relationalism as our theoretical framework. We then describe the transdisciplinary mixed-methods research approach. We present the empirical results based on a public online survey and audience interviews and interpret our findings regarding the diversity of human–nature relations. Lastly, we discuss ways in which human–nature relations are connected to sustainability-oriented mindsets and behaviors.

## Theoretical framework

**Fig. 1:**
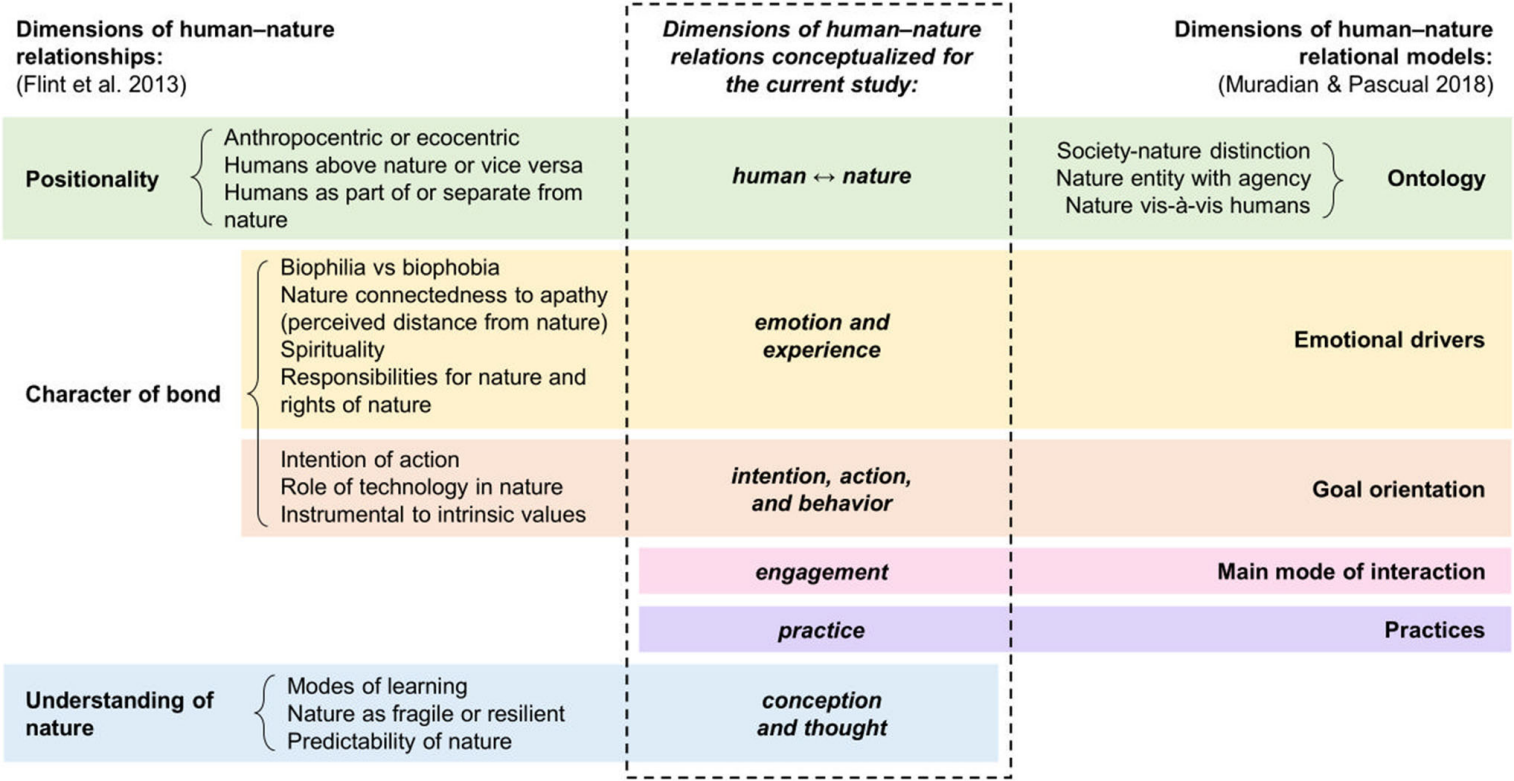
A theoretical framework indicating dimensions of human–nature relations for the purposes of the current study. The dimensions in the middle column were brought up in the transdisciplinary discussions among the authors and informed by literature (specifically Flint et al. 2013; Muradian and Pascual 2018). We interpreted empirical data into human–nature relations, expressed as discourses, and explored the characteristics of the relations using the above dimensions as an analytical tool

Discussions on human–nature relations are often laden with dualisms and divisionary debates. These easily oversimplify human–nature relations into single dimensions and highlight their opposite positions, such as ecocentrism vs. anthropocentrism or intrinsic vs. instrumental value (as discussed by, e.g., Ives and Fischer 2017; Manfredo et al. 2017; Bonnedahl and Heikkurinen 2019; Raatikainen et al. 2021). Yet, human–nature relations are diverse and dynamic, and we argue that there are several coexisting dimensions underlying this complexity. The existence of such dimensions can be inferred from the similarities in the verbal descriptions of human–nature relations. The similarities include, for example, notions of what is valued in nature, and how people position themselves in relation to nature. Various categorizations have addressed the dimensionality of human–nature relations, including ontological notions, ideologies, and material to philosophical connections with nature (Flint et al. 2013; Ives et al. 2017; Muradian and Pascual 2018). We have taken inspiration from earlier analytical frameworks on human–nature relations (Flint et al. 2013; Braito et al. 2017; Ives et al. 2017; Lumber et al. 2017; Muradian and Pascual 2018) to develop a theoretical lens suited for our research, which combines empirical research with relationalism (Fig. 1).

By examining conceptualizations, discourses, and experiences of nature, we set out to map different types of human–nature relations with the aim of positioning the empirical findings along theoretically defined dimensions of the relations. Here, a relational approach emphasizes that qualities of nature are not present in things but are derivative of relationships that occur directly between people and nature, or indirectly, i.e., between people but involving nature (Chan et al. 2016). In general, relationalism refers to an attempt to study social phenomena as fluid processes of interaction rather than solid, determining substances (Dépelteau 2018). Therefore, relationalism is well suited for exploring human–nature connections. Acknowledging that human–nature relations are in constant change helps to understand how they interact and influence each other (Ives et al. 2017).

As human–nature relations are constantly evolving, they hardly fit into fixed categories. There is some inertia in this process of becoming, though; human–nature relations are socially constructed and require shared cultural understanding to exist. To be able to co-create meaning for human–nature relations, people must share the verbal symbolism tied to the relations (Vandenberghe 2018). The relations are represented through language, conceptualizations, and manners of behavior. These evolve into discourses through repetition. Discourses portray shared ideas and guide practices in which different views of nature are embedded (Williams 1980). Subsequently, discourses encourage particular types of actions (Hugé et al. 2013). Thus, discourses encompass cognition and action as well as individual and collective perspectives. We argue that nature-related discourses provide a means to study the multidimensionality of human–nature relations, since discourses that differ content-wise (i.e., reflect different relations) can share similarities in their structure (i.e., underlying dimensions).

Flint et al. (2013) define three dimensions of human–nature relation: positionality of humans and nature, character of bond between humans and nature, and understanding of nature (see Fig. 1). This categorization was later used by Braito et al. (2017) to demonstrate how people’s behavior is connected to their individual nature relationship (how a person relates with nature). Actual interventions aiming at increased nature connectedness were studied by Lumber et al. (2017). According to them, experiential and affective factors such as perceived contact, emotion, meaningfulness, and compassion were predictors of connection with nature, whereas knowledge-based activities were not (Lumber et al. 2017). Ives et al. (2017) concluded that interventions targeting philosophical and emotional connections with nature had more potential to influence underlying values than interventions in cognitive, experiential, and material connections. Yet, values and other forms of cognition contribute to the emotional basis of interactions between people and nature (Jones et al. 2016; Ives et al. 2017), and the interactions turn into experiences that raise emotions (Raatikainen et al. 2020). Therefore, we wanted to examine also emotions and experiences, as well as actions and behaviors, as separate but interlinked dimensions with regards to conceptions and thoughts. In addition, we included two dimensions (engagement and practice) that have been highlighted as contributing to environmental behaviors (Muradian and Pascual 2018). By the dimension of engagement, we refer to the ways in which human–nature relations are concretized or operationalized (“main modes of interaction” sensu Muradian and Pascual 2018). Types of engagement include various forms, such as utilization and worship. The dimension of practice includes social rules, norms, and rituals that define acceptable actions and behaviors in relation to nature (Muradian and Pascual 2018). The other dimensions brought up by Muradian and Pascual (2018) corresponded to those of Flint et al. (2013) (Fig. 1).

Our analysis was inspired by the Maussian view on relational thinking that encourages a structural analysis of relations as a system of representations through which people are connected to each other (Vandenberghe 2018). In our case, nature-related discourses are such shared representations that are culturally produced in language. Communication in accordance with the discourses is a prerequisite for co-operation and collaboration, as any social action builds on shared meanings, norms, and values (Vandenberghe 2018). Yet, people give nature different meanings depending on the temporal, spatial, and social context (Williams 1980; Cronon 1996; Björklund et al. 2022), illustrating plurality in human–nature relations (IPBES 2022). Importantly, cultural representations of nature affect how people perceive nature and their own position in relation to nature. This has direct consequences on people’s worldviews and behavior (Castree 2001; Björklund et al. 2022; IPBES 2022). Therefore, by studying nature-related discourses we aimed to better understand the outcomes (actions, practices) of human–nature relations as well as their drivers (shared values and intentions), and the emotional and experiential factors that are important for individuals in deepening their nature connectedness and adopting pro-environmental behaviors.

## Materials and methods

### Data collection

**Fig. 2:**
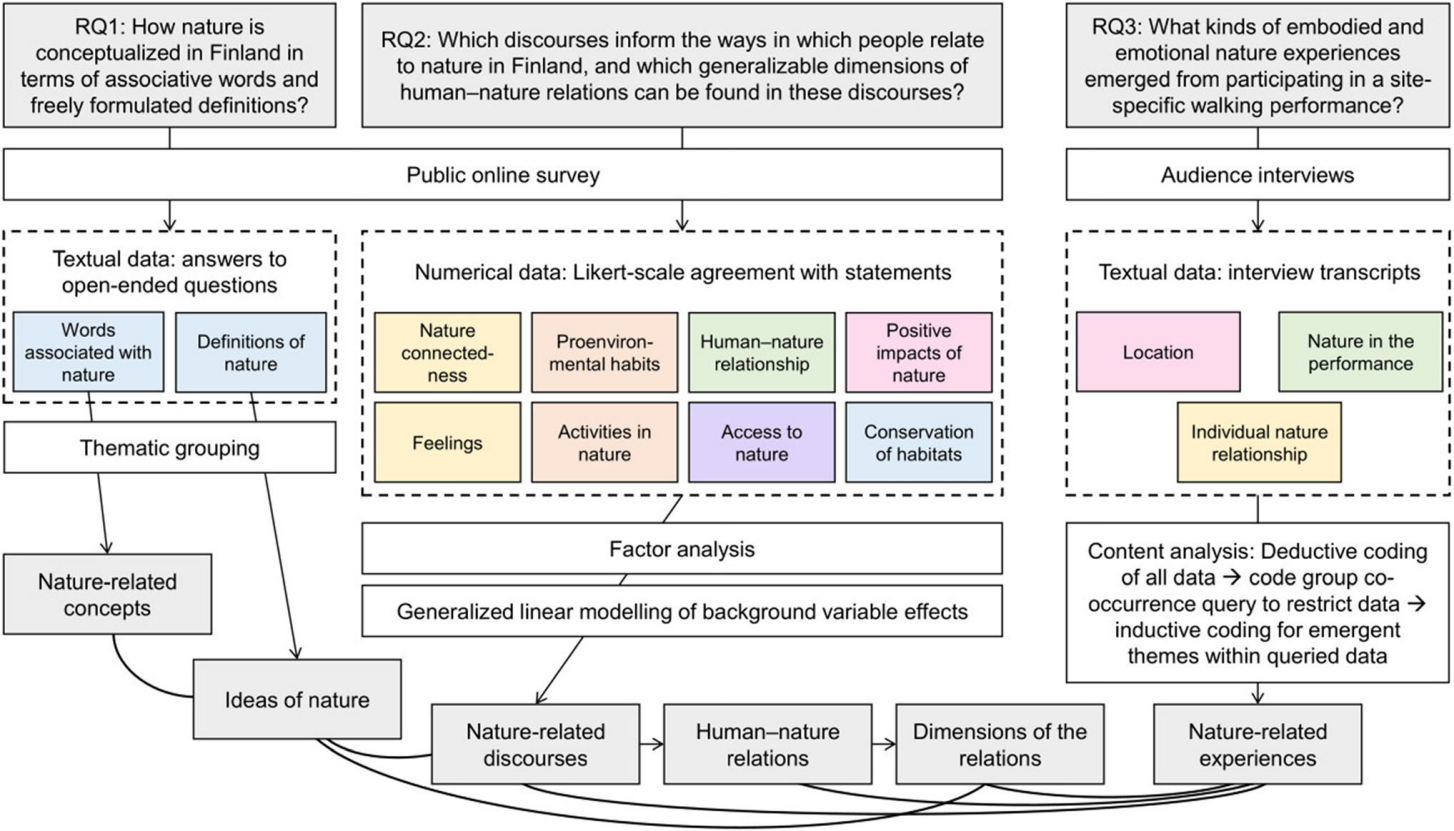
The mixed-methods approach of the study. Research questions and corresponding terminology used in Results section are in grey boxes. The white boxes refer to data collection (upper part) and analysis methods (lower part). Main data types are bounded with dashed lines, and colored boxes indicate data structure (sections in the survey and the interviews). The colors indicate connections with the theoretical dimensions of human–nature relations (Fig. 1). Arrows illustrate workflow, and the thicker lines at the bottom show comparisons between the results from the different analyses

According to a convergent mixed-methods design, we collected survey and interview data parallel to each other and analyzed them separately (Creswell and Creswell 2018). We then compared and synthesized the results of the analyses, assuming that a combination of different types of information would give a more detailed understanding on the diversity of human–nature relations in the Finnish context (Fig. 2).

We conducted a public online survey in between August 7th and November 2nd 2020, to collect qualitative and quantitative data on human–nature relations in the Finnish context (RQ1 and RQ2). The timing overlapped with the walking performance arranged on August 22nd and 23rd, 2020. The qualitative data collected through interviewing the audience was analyzed to explore the experiential aspects of human–nature relations (RQ3).

Details of the survey and sampling are provided in Appendix S1, and the questionnaire form is in Appendix S2. The anonymized survey dataset generated during the study is available in the JYX Digital Repository (Raatikainen et al. 2023). The following sections provide brief summaries of the analyses, which are described in more detail in Appendices S3 (thematic analysis on nature conceptualizations), S4 (factor analysis on shared nature discourses), S5 (respondents’ background), S6 (audience interviews), and S7 (code system for the deductive content analysis).

### Analyses on survey data

We analyzed textual data from two open-ended survey questions: respondents’ definitions of ”nature”, and the associative word lists by which they described nature. We analyzed these data by grouping the content of the responses under emergent themes (Appendix S3). The nature definitions were interpreted into ideas of nature that were shared by respondents, and the associative words were summarized into a condensed list of repetitive nature concepts.

Likert-scale numerical data was collected on respondents’ agreement with 84 nature-related statements. These data were analyzed using exploratory factor analysis (Appendix S4). Here we assumed that quantifiable patterns in respondents’ statement agreement and disagreement could be translated into shared discourses. The content of the discourses was interpreted based on statement associations within each factor and examination of statements with high loadings to each factor.

After a final set of six factors was formed, we calculated score values of each factor for every respondent. The factor scores indicated respondent’s agreement with the discourses, and we used them as response variables in analyzing the effect of respondent background on the discourses using generalized linear modelling (GLM; Appendix S5). In addition, we identified 20 respondents who had the highest scores for each factor. Their responses to open-ended questions were used to identify the connections between factor-based discourses (RQ2) and qualitatively interpreted nature conceptualizations (RQ1).

### Analysis on audience interviews

We applied arts-based methodology in the qualitative data collection. We interviewed audience members of a site-specific walking performance that was arranged at the Hitonhauta conservation area in Central Finland. The performance included a guided, silent walk trespassing the area. Eight acts were performed at different locations along the route, representing different aspects of human–nature relations in Finland. The arts-based research approach is described by Niemelä et al. (2023).

After the performance, the audience could voluntarily participate in a structured research interview (Appendix S6). At the onset of the interview, participants gave their informed consent and agreed with data collecting privacy policy and audio-recording. As the data collection included participation in public event, we did not collect participants’ background information to minimize the amount of personal data collected.

**Fig. 3:**
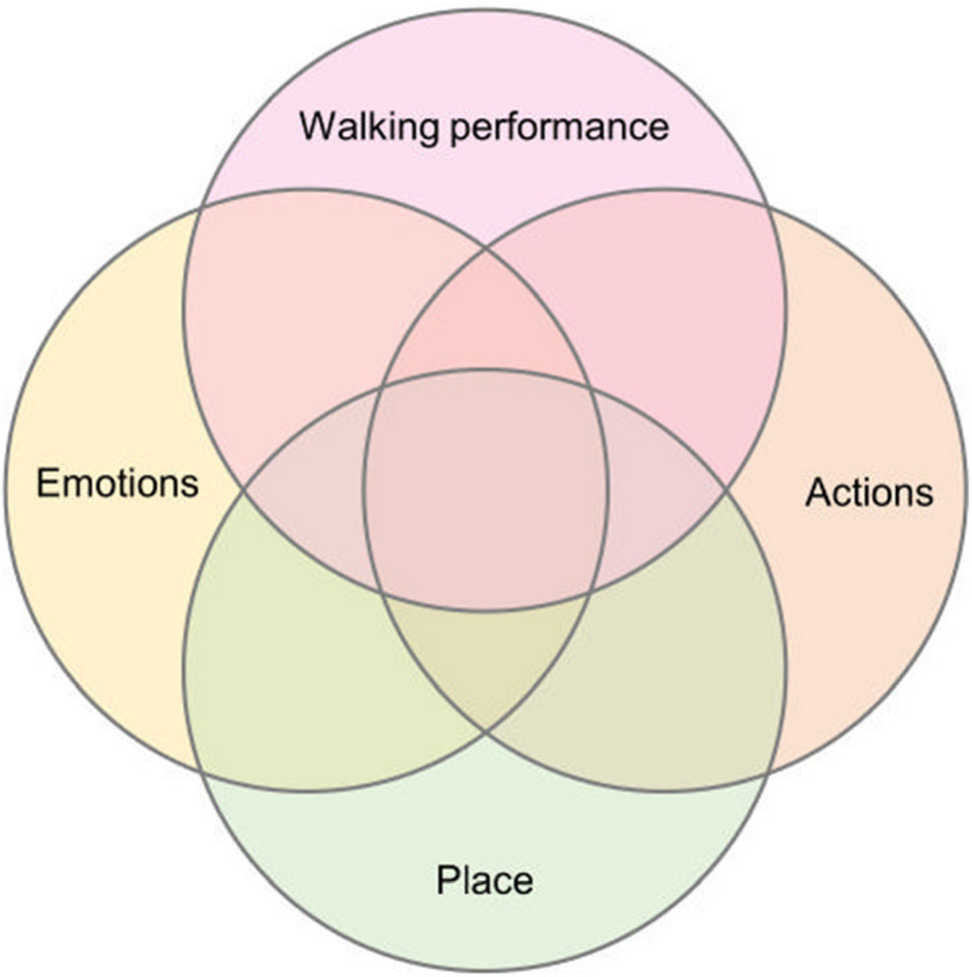
The interview data were queried to derive parts with overlapping content under selected code groups (i.e., areas covered with multiple circles). Extracted accounts indicated participants’ emotional reactions or embodied and sensorial experiences (code group Emotions) and content describing different actions and behaviors in nature (code group Actions), either of which coincided or were located near to codes under groups Place or Walking performance. Code group Place included participants’ observations on nature at the location such as habitats, weather, or landscape. Code group Walking performance included notions of the artwork

The interviews were transcribed verbatim and their content was qualitatively analyzed in two phases. First, we coded the transcripts deductively (Elo and Kyngäs 2008). We developed the code system (Appendix S7; Table 5) to detect which parts of the data were most informative according to RQ3. In the second phase of the content analysis, a query tool was applied on the coded data to search for overlapping and neighboring co-occurrences of code groups Emotions, Actions, Place, and Walking performance (Fig. 3). The derived parts of the data were coded again, this time using an inductive approach to detect emergent themes (Elo and Kyngäs 2008).

## Results

### Survey: Respondent characteristics

A total of 726 respondents participated in the survey. Responses were received from all 19 Finnish provinces. A total of 466 respondents (64.2%) lived in Uusimaa, Central Finland, South-Western Finland, or Pirkanmaa regions. All other provinces had less than 50 respondents each (2 to 47; median was 17 respondents). Seven respondents (1.0%) lived outside of Finland. Most respondents (82.1%) lived in an urban or village environment whereas rural inhabitants were fewer (17.9%). A typical respondent was a middle-aged, employed female with higher education, living in a city or village (Table 1).

**Table 1:** Respondent characteristics

Variable	Categories	N:o of respondents	% of respondents
Age class	18–24 years	29	4.0
	25–34 years	114	15.7
	35–44 years	168	23.1
	45–54 years	165	22.7
	55–64 years	145	20.0
	65 or older	105	14.5
Gender	Female	496	68.3
	Male	210	28.9
	Other	5	0.7
	NA	15	2.1
Level of education	Primary/grammar school	12	1.7
	Secondary school/junior high	30	4.1
	High school	53	7.3
	Professional training	222	30.6
	University or polytechnic	396	54.5
	NA	13	1.8
Employment	Entrepreneur	47	6.5
	Employee	394	54.3
	Not employed	131	18.0
	Retired	140	19.3
	NA	14	1.9
Living environment	Urban or village	596	82.1
	Rural	130	17.9
Type of home	House	282	38.8
	Rowhouse/condo	122	16.8
	Block/apartment	320	44.1
	NA	2	0.3

NA = Not applicable; respondents not answering the question. The percentage is calculated from the total number of respondents (*n* = 726)

**Fig. 4:**
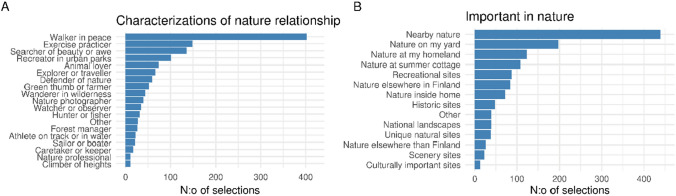
Respondents’ nature relationship characterizations (**A**) and features they considered most important in nature (**B**). Respondents could choose one or two options in both cases. The bars show the total number of selections for each option

Over half of the respondents visited nature frequently: either on daily basis (20.1%) or at least 3–6 times per week (37.3%). Every fourth (24.5%) reported 1–2 nature visits per week, and nearly every fifth (17.9%) responded that they did not go into nature that often. One respondent did not provide information on the frequency of nature visits. Respondents were also asked for one or two most common activities they did while being in nature. They most often were spending leisure time or exercising (527 and 490 replies, respectively). Peaceful walking within the everyday environment was the most popular way of engaging with nature, although other kinds of nature relations and priorities were also evident in the data (Fig. 4).

### Survey: Nature-related conceptualizations

We examined concepts and ideas associated with nature to understand how survey respondents conceptualized nature. When asked to describe nature, respondents (*n* = 666) listed a total of 3925 associative words that we interpreted into 132 underlying concepts. The most frequent concepts expressed by the respondents were peace and beauty, followed with integrity, greenness, and purity (Table 2).

**Table 2:** Nature-related concepts derived from associative words provided by survey respondents (*n* = 666). The concepts are grouped according to corresponding ideas of nature that summarize the respondents' nature definitions (*n* = 668), in the order of rough prevalence. Frequency refers to the number of associative words related to each concept, including words with shared root forms and/or meaning (see Appendix S3), as exemplified in the third column. To shorten the table, 43 concepts based on one to two mentions (freq. = 1–2) are omitted

Concept	Freq	Examples of words in data	Idea of nature as
Diversity	134	Diverse, diversity, varying, complex, different, forms	Ecological elements (24 related concepts)
Plants	100	Plants, vegetation, berries, moss, flowers, grass, photosynthesis	
Animals	96	Animals, birds, mammals, fishes, reptile, fauna	
Water	95	Water, waterways, lakes, sea, stream, wet, dew	
Forest	78	Forest, wooded, grove, spruce forest, pine forest, forestry	
Trees	60	Tree(s), deadwood, pine, spruce, birch, aspen, linden, oak, apple, cherry, resin	
Landscape	34	Landscape(s), view, picturesque, terrain, hill, ravines, mountain(s), countryside	
Mire	24	Mire(s), bog, fen	
Rock	21	Rock(s), cliff, block field	
Weather	20	Weather, rain, fog, storm, thunder, clouds, clearness	
Fungi	19	Mushroom(s), fungi	
Insects	18	Insect(s), bugs, butterflies, mosquitos, flies, horseflies, dragonflies, ants, ant hill	
Ground	13	Soil, ground, sediment	
Meadow	9	Meadow(s)	
Shore	9	Shore, beach, reedstand	
Field	8	Field(s), crops	
Fjell	8	Fjell(s)	
Organisms	8	Organism(s), species, biota	
Habitat	6	Habitat	
Climate	5	Climate	
Island	4	Island, archipelago	
Microbes	4	Microbes, bacteria, viruses	
Lichens	3	Lichen, beard moss	
Park	3	Park(s)	
Beauty	204	Beauty, beautiful, aesthetic, admirable, wonderful	Sensed and valued (21 related concepts)
Greenness	157	Green, greenness, lush, verdant	
Silence	69	Silence, silent, quiet	
Sound	62	Sounds, sighing of trees/wind, rustle, birdsong, waves lapping, water tinkling	
Scent	56	Scent(s), fragrant, smell	
Colors	45	Colors, colorful, blaze of color, fall colors, blue, purple, white, grey	
Closeness	33	Friend, close, own, personal, private, lap, approachable, pleasant	
Love	24	Dear, love, gentle, endearing	
Touch	21	Soft, warmth, cold, prickly, hard, hot, dry, feel	
Goodness	16	Good, goodness, nice, ok, positive, caressing	
Fear	14	Scary	
Light	14	Light, bright, dusk, sunshine, sun, moon, stars	
Senses	14	Senses, sensuous, perceptible, look, see	
Joy	12	Joy, gratefulness, pleasant, amiable, exhilarating, entertaining	
Worry	11	Worry, stress, restlessness, depression, solicitude	
Feeling	5	Emotional, feeling(s)	
Happiness	5	Happiness	
Ease	4	Easy, easily	
Hope	3	Hope	
Liking	3	Like	
Shadow	3	Shadow(s)	
Surprise	128	Surprising, unpredictable, miraculous, incredible, unknown, secret	Essence of life (13 related concepts)
Life	127	Living, life, vitality, vibrant, provider, biotic, organic	
Continuity	100	Age-old, eternal, time, continuous, permanence, origin, death, reproduction	
Strength	71	Strength, strong, empowering, force, powerful, energy	
Vastness	51	Space, open, infinity, vast, wide, high, large, deep, emptiness, sky, feeling small	
Being	36	Is, would, be, subsistent, present, presence, essential	
Inspiration	35	Interesting, fascinating, inspiring, invitation, calling, curiosity, creativity	
Richness	25	Richness, rich, abundant, abundance	
Balance	24	Balance, harmony	
Respect	18	Respect, respectful, majestic, mighty, sovereign, sublime, stunning, grandness	
Sacredness	17	Sacred, deity, temple, church, creator, devotion	
Spirit	7	Spirit, spiritual	
Wisdom	3	Wise	
Change	135	Changing, renewing, seasons, cycles, adaptation, growth	Living systems (7 related concepts)
Connection	51	Connection, connective, common, network, rootedness, impact, interaction	
Place	17	Place(s), site, location, region, area	
Action	15	Function, action, acting, actively, practicing	
System	9	Ecosystem, system	
Environment	7	Environment, surrounding	
World	4	World, earth, globe, Gaia	
Value	110	Important, crucial, priceless, valuable, intrinsic value, best	Wild and free (6 related concepts)
Wilderness	61	Wild, wilderness, untamed, uncontrollable, unoccupied	
formidability	60	Rough, harsh, austerity, dangerous, menacing, rugged, atrocious, merciless	
Freedom	56	Free, freedom, freely	
Vulnerability	47	Vulnerable, wounded, needing protection, threatened, fragile, sensitive, victim	
Wrongdoing	4	Undervalued, misunderstood	
Peace	337	Peace, peaceful, soothing, meditative, tranquil, rest	Source of wellbeing (5 related concepts)
Purity	155	Pure, purity, purgative, fresh, freshness	
Wellbeing	70	Refreshing, stimulating, healing, nurturing, therapeutic, wellbeing	
Safety	69	Safe, shelter, refuge, approval, familiar, home, mother, cradle	
Movement	14	Movement, wanderer, walk, roll	
Integrity	170	Independent, unworked, whole, entire, complete	Related to culture (5 related concepts)
Nature	50	Nature, natural, naturalness, organic, native	
Human	45	Human, people, us, mind, body, thought, observation, opinion, consciuos, intellect	
Meaning	5	Meaning, figurative, defining	
Particularity	4	Particular, details, detailed	
Giving	48	Give, giving, giver, generous, fulfilling, enabler, possibility, rewarding	Provider for people (5 related concepts)
Air	45	Air, oxygen, oxygenous, wind, atmosphere	
Breathing	17	Breath, breathing, breath-taking, lungs	
Nutriment	16	Nutriment, nutrition, food, nourishing, fishing, hunting, collectables, food chain	
Receiving	16	Benefit, use, want, need, demanding, productive, exploitable, ecosystem services	
Dualism	26	No-/not-/non-, other(s), outside, alien, dichotomy	Unbuilt environment (3 related concepts)
Path	14	Paths(s), routes, way, duckboards	
Building	3	Build, building(s)	

We derived nine broad ideas of nature from the respondents’ nature definitions (the right-hand column in Table 2). Two ideas (nature as “living systems” or “ecological elements”) represented a natural scientific approach and were cognitively grounded, but they differed in how they approached nature in epistemological terms. The idea of living systems defined nature as consisting of ecosystems and was clearly synthetic and process-oriented, representing principles of modern ecology; for example, “nature contains all biotic and also the abiotic things” (R551). The idea of nature as an assemblage of ecological elements was more atomistic and focused on observable entities in nature, reflecting the long-term heritage of taxonomy. Respondents characterized it with lists of species and habitats.

The natural scientific ideas took a value-neutral stance on nature, but this did not apply to the rest of the ideas. Overall, positive valuations of nature dominated in the data. We derived two ecocentric ideas that highlighted nature’s intrinsic value. One of these perceived nature as “wild and free” and represented strong normative claims towards untouched and undisturbed nature, defining it as “primordial, pure, unspoiled environment” (R464). The hard ecocentrism of wild and free nature was somewhat softened in the other idea, which we named as “unbuilt environment”. This latter idea conceptualized and valued nature as something outside of human influence: “Nature is what is outside of the walls. The thing that comes in if not kept away” (R185). Both ecocentric ideas were dualistic as they positioned people and nature against each other.

Nature was perceived as intrinsically valuable also in a non-dualistic sense. The “essence of life” idea adopted a holistic perspective that placed people as part of nature and highlighted spiritual and philosophical relations to nature. In this sense, nature was defined as the source and prerequisite of all life, people included: “I am part of nature. Nature is the foundation of all being” (R290).

We also found three ideas of nature that were inclined towards anthropocentrism. The first of these focused on the experience of nature connectedness. According to this idea nature was “sensed and valued” from a human perspective, and the deep meaning of nature for people, as well as the values people place on nature, were highlighted. Idea of nature as sensed and valued revealed the deeply interpretative character of nature conceptualizations. It even internalized nature into a bodily experience and a resulting state of mind. For example, nature was “experienced through different senses; landscapes and views; smell and touch, being in the middle of something larger and beyond control” (R256).

The other two anthropocentric ideas differed in their degree of utilitarianism. One of them defined nature as a “source of wellbeing” and perceived people as receivers of nature’s goods. Here people’s engagement with nature was described in passive terms: wellbeing was borne from peace and tranquility, and nature provided these by offering a place for rest. Typically, nature was defined “as a place to calm down and enjoy. Place where my soul and body get well” (R32). Nature as a source of wellbeing was contrasted with another utilitarian idea, which we named “provider for people”. Here, too, nature was seen as benefiting people, but people were described as active takers and co-creators of such benefits. Some accounts portraying nature as a provider for people brought up also material and physical connections between people and nature, including nutrition and work. One such example was a succinct definition of nature as “prerequisite of life” (R273).

Finally, we discovered an idea that defined nature as something “related to culture”. This idea approached nature as an oxymoron that was defined in terms of decreasing human influence while arising from deeply cultural origin. This conceptual fluidity revealed an intellectual struggle on what nature is: how its meaning can be constructed in various ways, and how the concept of nature exists because of its cultural context. For example, R226 defined nature as “a culturally constructed concept, the opposite of ‘culture’”, and R146 noted that “nature is always defined by people, it changes through time”.

When we compared the respondents’ nature definitions with their word lists, we observed diverse connections. Respondents combined several ideas and concepts in their accounts. R24 exemplified this by defining nature as “nearly everything you see when you walk out through the door. The terrain, sky, rivers and lakes, and animals”, and describing nature using words “peace, fresh air, diversity, primitiveness”. She conceptualized nature as consisting of items observed through senses, existing beyond the walls of one’s home, encompassing both abiotic and biotic variation, having mental and physical impacts, and under minimal human impact.

### Survey: Nature-related discourses

The factor analysis on nature-related statements found six discourses, each focusing on different kinds of human–nature relations (Table 3). The overall consistency of the statement data was high (Cronbach’s α = 0.94), thus indicating strong reliability. The cumulative overall variance explained by the six factors was 0.36, and the mean item complexity was 2.4.

**Table 3:** Factor properties. Discourses were named based on the interpretation of the factors. The numerical columns give key statistics for each factor (var. = variance). Associated survey topics give a general view on the factor contents, based on the statement-to-statement correlation matrix. Statements with polarized loadings exemplify key content for each discourse; the standardized loading is given in brackets. Note that negative loadings under ML6 indicate respondent disagreement with the listed statements

Factor	Discourse on nature	Initial eigenvalue	Sum of squared loadings	Proportion of overall var. accounted for	Relative amount of var. explained by the factors	Associated topics in the survey	Statements with polarized loadings
ML1	Wellbeing	18.06	6.57	0.08	0.21	Nature connectedness, positive impacts	Nature brings me joy (+ 0.73), nature invigorates me (+ 0.73), nature calms me down (+ 0.72)
ML4	Natural habitats	4.69	5.78	0.07	0.19	Conservation value of different habitats	Baltic Sea coast (+ 0.66), mires (+ 0.65), rock outcrops and scree (+ 0.65)
ML6	Ecoanxiety	3.01	5.73	0.07	0.19	Environmental concern, intrinsic value of nature	People are more important than nature (-0.54), I think environmental issues are exaggerated (-0.54), I want to see also human handprint in the landscape (-0.53)
ML3	Pro-environ-mentalism	2.11	5.33	0.06	0.17	Pro-environmental habits, environmental concern	I'm willing to pay more for environmentally friendly products (+ 0.64), I'm ready to compensate the harm I do to nature (+ 0.64), I'm ready to reduce car driving for environmental reasons (+ 0.52), I have to change my consumption habits for nature's benefit (+ 0.52)
ML5	Outdoor activity	1.59	4.15	0.05	0.13	Activities in nature, access to nature	I go into nature despite bad weather (+ 0.55), camping is the best part of my nature excursions (+ 0.51), going out to collect mushrooms and/or berries is important for me (+ 0.50), I like to go boating and/or paddling (+ 0.50)
ML2	Enjoyment	1.45	3.23	0.04	0.10	Positive impacts, sensing nature	The best things in nature are sounds, smells, sensations, or tastes (+ 0.61), the best things in nature are colors, views, or sceneries (+ 0.56), I forget my worries and troubles when I'm in nature (+ 0.43)

The most prevalent factor (ML1 wellbeing) reflected a discourse focusing on individual-level impacts of nature, including mental and physical benefits and health effects. It was followed by two factors that explained an equal amount of variance in the dataset. We interpreted these into a discourse tied to the overall conservation value of different kinds of natural habitats (ML4 natural habitats) on the one hand, and a discourse on strong environmental concern linked to intrinsic valuation of nature and human–nature dualism (ML6 ecoanxiety) on the other hand. The latter resembled the following factor (ML3 pro-environmentalism) due to its focus on environmental issues. A key difference between the two was the allocation of the more pessimistic and emotionally loaded statements into the ecoanxiety discourse, whereas the pro-environmentalist discourse focused on lifestyle- and solution-oriented topics.

We interpreted the final two factors into an action-oriented discourse (ML5 outdoor activity) and a discourse on the positive impacts of being in and sensing of nature (ML2 enjoyment). The importance of access to nature, and direct contact with nature, was evident in the outdoor activity discourse. Its emphasis was on *doing* whereas the enjoyment discourse highlighted *being* in nature. The enjoyment discourse shared content on the restorative impacts of nature with the wellbeing discourse, while focusing more on the transient character of nature experience. The wellbeing discourse spoke more of consequences of nature contact when compared to the enjoyment discourse.

The GLM analyses provided information on how the respondents’ background affected their relatedness with the discourses (Table 4). The wellbeing discourse was tied to an increasing number of nature visits, and this effect was similar in all age classes. Contrastingly, two other discourses were connected to respondent age but not to the frequency of visiting nature. The natural habitats discourse, which highlighted targets of conservation effort, was more common among older respondents, whereas younger respondents had higher relatedness with the ecoanxiety discourse. The discourses on pro-environmentalism and outdoor activity were more prevalent among younger respondents, as well as among those respondents who visited nature more often, thus expressing an interaction between respondent age and frequency of nature visits. Finally, the enjoyment discourse was indifferent according to both respondent age and the frequency of nature visits.

**Table 4:** Results of generalized linear models (GLMs) on factor scores. SE = standard error. Statistically significant (Sig.) coefficients are denoted according to the p values: *** < 0.001; ** < 0.01; * < 0.05. Note that in log-linear GLMs the regressor coefficient estimates represent multiples of change in the response variable

	Coefficients	Estimate	SE	t value	p value	Sig
ML1 wellbeing discourse:						
	(Intercept)	1.7223	0.0224	77.03	< 2 × 10^−16^	***
	Respondent age	0.0003	0.0004	0.82	0.411	
	Freq. of nature visits	0.1047	0.0181	5.78	1.10 × 10^−8^	***
ML2 enjoyment discourse:						
	(Intercept)	1.7715	0.0228	77.83	< 2 × 10^−16^	***
	Respondent age	0.0006	0.0004	1.39	0.164	
	Freq. of nature visits	− 0.0141	0.0185	− 0.76	0.445	
ML3 pro-environmentalist discourse:						
	(Intercept)	1.8112	0.0225	80.53	< 2 × 10^−16^	***
	Respondent age	− 0.0011	0.0004	− 2.64	0.008	**
	Freq. of nature visits	0.0643	0.0184	3.50	0.001	***
ML4 natural habitats:						
	(Intercept)	1.6997	0.0225	75.39	< 2 × 10^−16^	***
	Respondent age	0.0018	0.0004	4.45	9.89 × 10^−6^	***
	Freq. of nature visits	0.0103	0.0182	0.57	0.572	
ML5 outdoor activity:						
	(Intercept)	1.7745	0.0209	84.82	< 2 × 10^−16^	***
	Respondent age	− 0.0016	0.0004	− 4.10	4.52 × 10^−5^	***
	Freq. of nature visits	0.1803	0.0171	10.55	< 2 × 10^−16^	***
ML6 ecoanxiety:						
	(Intercept)	1.8802	0.0224	84.08	< 2 × 10^−16^	***
	Respondent age	− 0.0017	0.0004	− 4.24	2.48 × 10^−5^	***
	Freq. of nature visits	− 0.0090	0.0184	− 0.49	0.625	

**Fig. 5:**
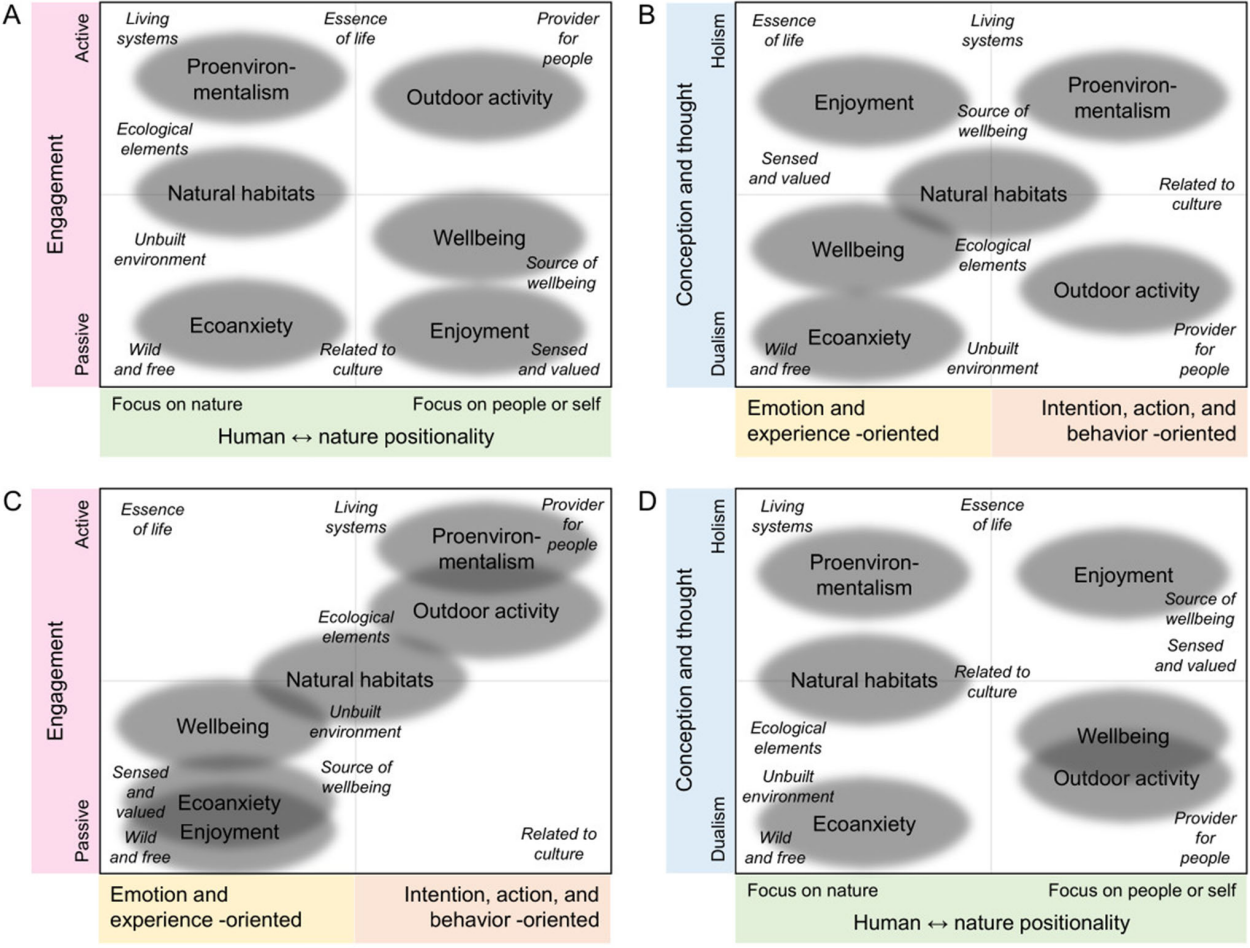
Interpretation of the survey results according to theoretical dimensions of human–nature relations. Grey ovals represent nature-related discourses and nature ideas are shown in *italics*. The positioning of the discourses and ideas is based on their content in relation to the dimensions shown (**A** nature- or people-centered positionality vs. passive to active engagement; **B** emphasis either on emotion and experience or intention, action, and behavior vs. dualistic or holistic conception; **C** and **D** show alternative axis arrangements). The proximity of the discourses and the ideas indicate close connection; however, the actual distances of the items are not explicit since their placement along the dimensions is indicative. For example, in panels **A** and **C**, people’s active engagement with nature is depicted as a prerequisite for ideas of nature as essence of life, provider for people, and living systems (of which people are part of). These are contrasted with ideas of nature that display people’s role as more passive (nature as sensed and valued), abstract (related to culture), or undesirable (wild and free)

Based on the high-scoring respondents’ nature definitions we observed that several ideas of nature were connected to each discourse. For example, the wellbeing discourse was linked to ideas of nature as a source of wellbeing, related to culture, unbuilt environment, and provider for people. We identified the most prevalent ideas based on the respondent accounts and discourse content and compared these interconnections to the dimensions compiled in our theoretical framework (Fig. 1). The resulting graph provides an overview on the observed dimensions and the focal contents of human–nature relations (Fig. 5). Figure 5 also illustrates how the similarities and differences among the relations become visible when they are placed according to different dimensions.

### Audience interviews: overall content

Of the 140 persons attending the site-specific walking performance, 71 persons participated in research interviews. The interviews portrayed a further diversity of ideas of, and attitudes towards nature, and indicated that participation in the performance focused participants’ attention to a variety of sensory and embodied experiences as well as to the particularities of the location (Niemelä et al. 2023). Participants reflected on the performance and their nature experience, bringing up also topics relating to emotions, memories, philosophical ideas, and wellbeing.

The interviews were able to capture the emotional aspects of attending the performance. The code group Emotions had the highest frequency of coded data segments, containing participants’ emotional reactions and sensory experiences in relation to the performance and the site, as well as their memories of earlier visits there (Table 5). Other two frequent code groups were Walking performance, containing mentions regarding the performance, and Place, containing observations on the location, such as weather events, biotic and abiotic elements, and nature as stage of the performance.

**Table 5:** The code groups (header), codes and their frequencies (columns) in the interview data collected in the occasion of the walking performance (*n* = 71). The frequencies indicate the number of quotations under each code. The overall content of the interviews was deductively analyzed using six code groups and 58 codes (Appendix S7). The codes denote observations made by the interviewees on either the performance or the surrounding nature, and nature-related conceptualizations, themes and topics brought up in the interviews. Different parts of the performance are categorized as acts under the code group Walking performance

Code group:	Place	Freq	Walking performance	Freq	Views and conceptions	Freq	Emotions	Freq	Actions	Freq	Human–nature	Freq
Codes:	Hitonhauta gorge	84	Performance in general	194	Nature conservation	41	Emotions	88	Perceiving	67	Agency of nature	52
	Nature as a stage	71	Conservation of Nature (act)	36	Health effects	30	Sensory experience	73	Staying still	30	Humans as part of nature	37
	Biotic nature	49	Antagonistic Nature (act)	34	Utilitarianism	28	Interpretations of the performance	72	Rights to roam	28	Generations	29
	Weather, seasons	49	Mother Nature (act)	34	Cultural traditions	24	Experiencing awe in nature	51	Being in silence	28	Respect towards nature	29
	Abiotic nature	46	Mythic nature (act)	26	Religiousness	21	Experiencing a sense of fracture	50	Feeling harmony	15	Feeling small in face of nature	11
	Landscape	38	Transitions (walk)	24	Mythology	19	Bonding with nature	47	Pace of the walking	14	Geological time	10
	Habitat types	32	From Spirits of Forest to Forest Industry (act)	22	Nature as a retreat	17	Embodied experiences	38	Group and group dynamics of a certain act	9	Humans as separate from nature	7
	Path	20	Cycles of Nature (act)	19	Value choices and value diversity	14	Being impressed by the performance	29	Nature contradiction recognized	8	COVID-19	5
	Space	16	Logger of the Trees (act)	17	Nostalgy	4	Sacredness, spirituality	22	Customs and habits linked to nature relation	6		
	Sense of place	9	Historical eras (act)	12			Memories	21	Livelihood as a mode of utilitarianism	1		
			Uniqueness	7								
Total freq.:		414		425		198		491		206		180

The rest of the interview content covered diverse aspects on human–nature relations. Participants reflected on actions and behavior in nature, including activities during the performance and their own habits and preferences to spend time in nature (code group Actions). Perceiving one’s surroundings and staying still, being in silence and grounded in the moment, were often mentioned. Participants elaborated various ideas of nature and described individual-level and societal relations to nature (code group Views and conceptions). For example, cultural expressions of nature relations ranged from contemporary conservation practices to traditions rooted in mythology. Participants’ reflections on the positionality between humans and nature included, for example, notions of nature’s agency, people’s dependence on nature, and respect towards nature (code group Human–nature). Regarding the dimensionality of the human–nature relations, the content of the interviews supported the survey results. However, the qualitative analysis enabled us to acquire more detailed insight into the experience of being connected with nature.

### Audience interviews: Embodied experiences

To examine the embodied aspects of human–nature relations, we explored nature-related experiences reflected by the participants (*n* = 71). The inductive content analysis targeted a specific part of the interview data, indicated with co-occurrences among code groups Walking performance, Place, Emotions, and Actions (see Fig. 3). We found five categories of nature-related experiences that emerged from the audience members’ accounts:

1. Sensory experience
2. Sense of connectedness
  - human–nature connection
  - human–human connection
  - connection to self
3. Feelings and other inner experiences
4. Values and norms
5. Sense of place

Each category described a different type of experience. The first group, sensory experience, included ways of being, perceiving, moving, and halting: staying still, in silence, focusing on the moment, and slow pace of walking. The sensory experiences were individual; based on participants’ physical presence in nature and mediated through their senses:

”Those things that I search for in nature were strongly present in the performance. In particular that tranquility, calming down, all that minimization of the sensory stimulus; it was built in the performance in a fine manner.”

Although the participants attended the performance as individuals, they experienced connection to nature, other participants, and themselves. We divided the sense of connectedness into three sub-categories depending on the direction of the experienced bond. Human–nature connections included accounts on interactions between people and nature, including those spanning over generations:

”I think that the performance was truly appealing, the cultural heritage was included and the way in which nature has been part of people’s life; how nature impacts people and all the good things nature does for us, how we are of nature.”

The social experience involving human–human connections became important during the walk, as the audience tackled the rough terrain together. The participants felt being connected to one another, and involved in the group:

”Everyone had to walk that rough path. In a way it started already in the beginning when we arrived at the setting. A small group of people, strange to each other, were sitting in the trailer of the tractor, and we saw only the treetops and the sky over the sides of the trailer. And then we landed and went through the terrain that was almost impassable to some. Everyone brought their bodies through it with their own strength and skills.”

The above quotation exemplifies the physical character of the experience: the participation required strength. This bodily connection to nature was accompanied with a consciousness of self and one’s capability to move in nature, denoting how the participants experienced independence while belonging to the group.

Participation in the event raised a range of feelings and other embodied and inner experiences, as the richest category according to the number of codes was Emotions. The participants expressed, for example, experiences of awe, calmness, groundedness, healing, and being safe:

”Some acts felt like they were made for me, they allowed me to look at myself as if from the outside; through the performers and the nature. There was that huge rock wall, it was exactly like the feelings that I’ve been going through lately.”

“…these people who performed with and without words, all this somehow nourished the experience and the nature made it much stronger. If I had seen these acts in a theatre, I might not have reached these same feelings. Here I could just curl up to myself in the lap of nature and it all went straight into emotions. It was amazing.”

The cultural aspects of engaging with nature were brought up by those participants who discussed values and norms in relation to their experience. Practices of working with nature and Finnish manners of relating to nature were mentioned. The topics ranged from traditions and memories to conservation actions and the global ecological crisis. Nature was approached as a place of harmony and wellbeing, as well as a source of livelihoods, material benefits, and wealth. The influences of the cultural interpretations of nature were discussed, as well as ideological changes:

”The [ecocentric] monolog in the end spoke of things that I already had started to think about: how this industrial world has gone out of control and we have lost our connection to nature; I guess it is the idea of control over nature, […] that idea has been a mistake and now we see how our relations to nature have changed.”

In general, interacting with nature was seen more intimate in the past than today:

”I was thinking about traditions and nature and how [they were entangled in the performance] — there were the blowing horns of the underworld, how the washing of the deceased was done, and all that.”

During the performance, the participants followed the path to and across the Hitonhauta gorge and experienced the place through their senses. They noted how the location played a key role in the performance. Their experienced sense of place included observations of nature as a stage, and how the performance, location, and nature seemed to merge:

“I thought that the performance was really made to this place, as it felt like the acts were growing from the sites where they were set. It was not just a performance brought to nature but a performance growing from nature.”

Participants contemplated also on nature’s agency and participation in the performance, and their accounts show how nature took the leading role, leaving the human performers aside:

“In many of the acts the human figure sort of lost its meaning.”

“[Nature] was not just the frame and the place but the performance connected people with nature. Now, when I think about it, I would say that this was what the performance was about: it showed the place of humans in nature, as part of nature, in every act and scene.”

Overall, the accounts of the performance audience highlighted the diversity of experiences of nature. This richness was apparent in the manifold reflections provided by the interviews.

## Discussion

### Human–nature relations in Finnish context

Our results demonstrate a diversity of human–nature relations coexisting within a relatively restricted context. In contemporary Finland, nature is largely viewed positively and considered important for the quality of people’s life. Qualities such as peacefulness, beauty, and integrity are often associated with nature. People spend time in nature, and are interested in nature-related matters. The awareness of environmental issues reflects a widespread concern over nature’s state. These overall findings are in line with two recent national surveys that also examined nature relationships in Finland (Sitra and Kantar TNS 2021; Finnish Environment Institute 2022, *n* = 2245; *n* = 1057, respectively). However, our analysis revealed a broader range of views of nature and illustrated also discrepancies among human–nature relations, supporting Björklund et al.’s (2022) argument that a conception of a single type of “Finnish nature relationship” is misleading. Furthermore, we were able to unveil the intricate connections among concepts, ideas, discourses, and experiences that underlie human–nature relations in Finland.

Human–nature relations change through time (e.g., Williams 1980). Thus, it is important to acknowledge that multiple conceptions of nature coexist (Björklund et al. 2022; IPBES 2022), and approach nature as an evolving concept (Castree 2001). We found nine dominant ideas of nature that exemplify how nature conceptualizations are rooted in context and cultural legacies. These contexts include scientific disciplines such as ecology and taxonomy, values placed on nature (Chan et al. 2016; Jones et al. 2016), and ideologies such as admiration of the wilderness (Williams 1980; Cronon 1996). The dynamism of human–nature relations maintains a situation in which people adopt parallel worldviews (e.g., IPBES 2022). We observed how the wilderness-oriented dualistic separation between people and nature can be accompanied with holistic ideas of nature as the essence of all life, people included, as well as more recent systemic views on nature, both ecological and social-ecological. Such intermingling of worldviews means that diverse approaches are needed to mainstream pro-environmental mindsets and behaviors that support sustainability (Braito et al. 2017; Ives et al. 2017; Muradian and Pascual 2018; IPBES 2022).

We approached human–nature relations empirically as structured systems of representations reflected in nature-related discourses (Hugé et al. 2013; Vandenberghe 2018). Our analysis revealed six different nature-related discourses that are common in Finland. The dominant discourse emphasized mental and physical wellbeing and health benefits derived from nature. The wellbeing discourse emerged also in the results of our qualitative analyses, according to which nature provided wellbeing for people in multiple ways, including offering peace and tranquility. Similar results were acquired in other recent studies (Sitra and Kantar TNS 2021; Björklund et al. 2022; Finnish Environment Institute 2022), indicating that the wellbeing discourse is strong in Finland.

We distinguished also other contemporary topics that characterized the observed nature-related discourses. Specifically, our results portray a multitude of views in relation to environmental issues and conservation needs. Conservation of natural habitats, ecoanxiety, and pro-environmentalism formed independent discourses. Under the conservation discourse, the respondents gave nearly equal value to all mentioned habitat types. Surprisingly, they did not prioritize forest conservation, which has for long been the dominant topic in the heated conservation discussions in Finland (Berglund 2001). The pluralization of conservation priorities may follow the new information provided by the assessments of threatened habitat types during the 2000s (Raunio et al. 2008; Kontula and Raunio 2018).

Environmental degradation including pollution and littering, climate change, and biodiversity loss are considered as important threats to Finnish nature, and over half of Finns are willing to take action on nature’s behalf in their everyday life (Sitra and Kantar TNS 2021; Finnish Environment Institute 2022). Our results support earlier findings, but allow for a more nuanced interpretation. The separation between the ecoanxiety discourse and the pro-environmentalist discourse demonstrates how differently people view the ecocrisis and their role in it. There were clearly two main approaches: that of pessimistic and passive respondents who emphasize the severity of the crisis and feel burdened by their inability to halt the detrimental trajectory (the ecoanxiety discourse), and that of more optimistic respondents who held hope that their actions matter and were thus more willing to act (the pro-environmentalist discourse).

We did not find evidence for a nature-related discourse based on apathy (i.e., human–nature relation seen as unimportant or unrecognized; Braito et al. 2017). This may be due to sampling bias, as the survey respondents and the interview participants were interested in nature-related matters and this clearly motivated their participation in the research. Yet, with the combined cumulative evidence of other recent research on Finnish nature relations (Sitra and Kantar TNS 2021; Björklund et al. 2022; Finnish Environment Institute 2022), we are rather confident in arguing that apathic or dismissive attitudes towards nature are rare in contemporary Finland. However, we observed that the ecoanxiety discourse may hold similarities with nature-related apathy as its inherent pessimism may paralyze pro-environmental action, even though the environmental crisis is at the heart of ecoanxiety.

In our data, the preferred discourse in terms of sustainability was the pro-environmentalist one. However, it lacked prominence when compared to wellbeing, natural habitats, and ecoanxiety discourses. Also content-wise, the dominant discourse on nature-derived wellbeing lies far from pro-environmentalism (Fig. 5). This indicates potential in enriching wellbeing emphasis with pro-environmentalism and vice versa. One way to strengthen the connections between different discourses is to communicate their content using the nature-related concepts and ideas that are common to various discourses. Specifically, the passive and anthropocentric tones of the wellbeing discourse would benefit from introducing action-orientation and ecocentric ideas. Here the idea of nature as consisting of ecological elements, combined with the familiar positive attributes of nature including purity, beauty, diversity, and intrinsic value, can act as an effective mediator.

In addition, we discovered human–nature relations building either on active doing (outdoor activity discourse) or sensing and being present in nature (enjoyment discourse). The content of these discourses and our qualitative findings on embodied nature experiences support results from parallel studies that contact with nature fosters the feeling of nature connectedness (Lumber et al. 2017; Sitra and Kantar TNS 2021; Finnish Environment Institute 2022). Overall, survey respondents considered themselves as frequent nature visitors. Their most common activities were walking and exercising, and they held easy access to nature important. In Finland, as in other Nordic countries, the rights to roam have allowed people to freely access nature for centuries. This cultural practice adds ease to being in contact with nature. Frequent nature visits supported the wellbeing, pro-environmentalist, and outdoor activity discourses. We discovered also further connections between the outdoor activity and pro-environmentalist discourses: both adopted an action-oriented stance and supported active engagement with nature (Fig. 5). We argue that synergies between these two discourses hold perhaps undervalued potential in mainstreaming sustainability. Recreational activities can be resource-intensive, specifically if they involve long-distance travelling, and pro-environmentalistic frugality would reduce their ecological footprint.

Although the enjoyment discourse was not statistically linked with visiting nature, the importance of experiencing connection with nature to enjoy it was evident in our qualitative data. This emphasizes the importance of pluralistically enhanced nature contact in strengthening nature connectedness (Lumber et al. 2017). We argue that emotional experiences grow often unconsciously from contact with nature and suggest that once nature attachment is emotionally formed, it persists even in the absence of recurring nature contact. Given that a person constitutes her/his relation with nature through interactions, cumulative nature-related experiences are important in the process. All experiences are mediated by senses and thus human–nature relations build on sensual events which feed into various conscious and unconscious processes within the human mind–body. This highlights how people are not aware of all ways in which they relate to nature, and the changes in human–nature relations may take time. A further aspect that emerged from the qualitative data was the sociocultural character of experiencing nature. Participation in a group that engaged with nature clearly added depth to the experience.

The sociocultural character of human–nature relations, which some survey respondents reflected under the idea of nature being defined in relation to culture, was positively emphasized by the participants of the walking performance. This illustrates how diverse human–nature relations coexist, and this complexity and contradicting values were central to the arts-based research method (Niemelä et al. 2023). Overall, the interviews illustrated similar discourses and respective human–nature relations compared to the survey, while adding experiential, embodied, philosophical, and over-generational aspects. The performance drew elements from mythology, traditions, history, religion, and ideologies, and some participants identified with these during the interviews. Wilderness-oriented accounts were fewer; in general, only few participants raised concerns over the fact that the performance took place on a conservation area, a concept that is in stark contradiction with the wilderness ideology and most conservation practices. Instead, many participants mentioned that the performance conveyed respect towards nature in diverse ways, eliciting wonder, gratitude, and environmental awareness among the participants.

### Dimensions of human–nature relations

We examined the structural characteristics of nature-related discourses to gain information on the dimensionality underlying human–nature relations (Figs. 1 and 5). Our analysis revealed three main dimensions within the discourses: positionality of humans and nature (ranging from ecocentrism to anthropocentrism), engagement (assuming either active or passive role of people), and conception and thought (cognitive approaches ranging from dualistic to holistic). The human–nature positionality appears as a fundamental dimension in human–nature relations as it reflects the way nature representations tie into people’s worldviews (Williams 1980; Castree 2001; Flint et al. 2013; Muradian and Pascual 2018). Positionality, engagement, and conception are also central in the life frames approach (living from, with, in and as nature) introduced in the IPBES values assessment typology (2022).

Our results link also to other relational dimensions, although they may not be as evident. The latent character of dimensions of emotion and experience; intention, action, and behavior; and practice does not imply unimportance. Rather, they may indicate deeply rooted aspects of human–nature relations that can be hard to examine (Ives et al. 2017). The dimension of nature-related practices was least evident in our data, yet it can be seen as the sociocultural ‘glue’ connecting other dimensions to each other, as exemplified by the earlier example on accessing nature using the rights to roam. As we studied nature-related practices in a very limited manner, we suggest they deserve more research using the relational lens.

Our qualitative inquiry illustrated that information on embodied and emotional nature experiences can be reached through arts-based research methods. The audience interviews from the walking performance revealed five categories of nature-related experiences that are connected to other relational dimensions such as human–nature positionality, engagement, and conception and thought. The experiential perspective highlighted the importance of senses in human–nature relations. Sensing and interpretation of sensory information into experience are basic functions of any living organism, and therefore they underlie all organism–environment relations, people included. For people, constructions of reality are always emotionally mediated, as emotions tint all human experience (Tuan 1977). Therefore, it is not surprising that the experiential and emotional character of human–nature relations has lately received attention in research on nature connectedness (e.g., Lumber et al. 2017; Raatikainen et al. 2020). The experiential interactions that provide ground for human–nature relations are place-bound (Tuan 1977; Schroeder 2007), which was elaborated by the participants’ and the survey respondents’ accounts. Based on the premise that place-based approaches support sustainable communities (Horlings 2015), relations between people and specific places are increasingly studied by sustainability scientists (Balvanera et al. 2017; Ives et al. 2017). The role of place-based experience in human–nature relations is an important topic for participatory and transdisciplinary research.

There is ample evidence that societal goals, values, and intentions shape human–nature relations (Flint et al. 2013; Ives et al. 2018; Muradian and Pascual 2018; IPBES 2019, 2022). These large-scale drivers are notoriously difficult to intervene (Ives et al. 2018). In Finland, utilitarian approaches to nature have dominated in politics and policy-making since the World War II (Björn 2000; Berglund 2001; Björklund et al. 2022). Utilitarianism continues to impact human–nature relations as long as nature is perceived as a source of raw materials and wealth. Our study did not differentiate actively utilitarian human–nature relations, but rather integrated utilitarian elements in the anthropocentric discourses, for example through the idea of nature as a provider for people. This dilution of utilitarianism was evident also in the interview accounts, where immaterial dependencies of people from nature were discussed more often than material benefits. We observed a similar process of attitudinal softening in relation to the wilderness ideology, as its abrupt, dualistic and ecocentric form was accompanied with softer ideas more inclusive of people’s handprint in nature. It is possible that such diversification is consequential for a general tendency to approve more pluralism in the Finnish society.

## Conclusions

We have illustrated the complex character of human–nature relations. Human–nature relations are borne out of interactions between people and nature; they thrive in all social levels from individual to cultural; take various coexisting forms that are not always compatible with each other; and influence the ways people perceive the world and place themselves in the world. All humans belong to societies, and all societies exist in relation to their environment. Because of this interdependency, human–nature relations manifest social and cultural structures that greatly influence the environment, ecosystems, and the Earth system. Therefore, both the causes of the ecocrisis and the opportunities to solve it are rooted in human–nature relations.

Considering the urgent need to halt climate change and biodiversity loss, the observed diversity of human–nature relations is challenging. It emphasizes that there are no blueprint solutions for promoting pro-environmental behaviors (IPBES 2022). As people value nature in diverse and sometimes conflicting ways, the debates around human–nature relations will continue to exist. From the sustainability perspective, these include conflicts between conservation and land use, ethical issues, and the ways in which nature is valued within societies (e.g., whether the ecological basis of human societies is substitutable with other types of capital; see Bonnedahl and Heikkurinen 2019).

The fact that pluralism may cultivate conflicts means that the diverse human–nature relations need to be better understood and communicated (IPBES 2022). If only selected kinds of human–nature relations are included in public discussions and environmental policies, just solving of environmental issues and the conflicts around them remains impossible. Undeniably, achieving sustainability requires wider adoption of approaches that respect and value nature, among which pro-environmental human–nature relations are portrayed as preferable. Our results show that there are multiple relations that can benefit nature. In fact, through dimensional similarities, human–nature relations that seem distant to each other may influence one another and evolve in unison. According to our results, the similarities that connect different kinds of relations include compatible views on human–nature positionality, practices of engagement, action-orientation in environmental matters, emotional connectedness to nature, as well as understanding of nature. These synergies need further exploration in research and can be cultivated in education and public discussions. It is important also to understand better which aspects of human–nature relations are at odds with each other and why; one example is the ecocentrism of the proenvironmentalist discourse that contrasts with the anthropocentrism of the outdoor activity discourse. Although these two discourses support each other in how they value nature and promote action, the positional difference may inflict discrepancies. Thus, sustainability transformation should not be advanced in a single-minded manner but proactively, building on a range of human–nature relations and using their malleability to bring different nature-related discourses closer to each other.

Due to the dynamic character of human–nature relations, new perspectives will come into effect. A prominent example of this is the wellbeing discourse, which has gained traction in Finland during recent years. The recent surveys clearly demonstrate its strength. The wellbeing discourse appreciates people’s sense-based and emotional attachment and attunement with nature, which opens a new venue to promote sustainable mindsets through self-reflection, action, and time spent in nature. The possibility to access nature, preferably in near environment, is key in supporting this; and arts-based practices provide novel tools to support people’s emotional and embodied connections with nature.

### Supplementary Information

Below is the link to the electronic supplementary material.

Supplementary file1 (PDF 333 kb)
