# Supplementary material for: The intricate diversity of human–nature relations: Evidence from Finland
Source: Ambio. 2023 Sep 29;53(2):181–200. doi: 10.1007/s13280-023-01933-1 (PMC10774321; doi:10.1007/s13280-023-01933-1)
Supplement: Supplementary file 1 — Supplementary file1 (PDF 333 kb) [file 13280_2023_1933_MOESM1_ESM.pdf]

Ambio

Electronic Supplementary Material

Title: **The Intricate Diversity of Human–Nature Relations: Evidence from Finland**

Authors: Kaisa J. Raatikainen, Anna-Kaisa Tupala, Riikka Niemelä, Anna-Mari Laulumaa

Contents

Appendix S1: Survey and sampling 2
Appendix S2: Questionnaire form of the online survey 2
Appendix S3: Thematic analysis on nature conceptualizations 10
Appendix S4: Factor analysis on shared nature discourses 10
Appendix S5: Respondents' background 11
Appendix S6: Audience interviews 13
Appendix S7: Code system for the deductive content analysis 13
References 16

Appendix S1: Survey and sampling

We conducted a public online survey under the title My Nature Relationship. The survey was targeted to Finnish-speaking adults, and it was open in between August 7th and November 2nd, 2020. The timing overlapped with the walking performance arranged on August 22nd and 23rd, 2020. The questionnaire form, written in Finnish, was created using Webropol 3.0 and consisted of 21 questions in 11 sections (web pages; see Appendix S2). The first section contained questions related to the respondent's background and was followed by two voluntary sections in which respondents replied to general questions on their nature relationship. The rest of the questionnaire form presented 84 nature-related statements. We placed statements derived from different contexts into eight sections (Fig. 2 in the article). The statements were grounded in literature on human–nature relations (Williams 1980; Cronon 1996; Haila 1999; Kellert 2006; Flint et al. 2013; Braito et al. 2017; Muradian and Pascual 2018), conservation (Ives and Fischer 2017; Manfredo et al. 2017; Kontula and Raunio 2018; Sandbrook et al. 2019), and sustainability (Ives et al. 2017; Lumber et al. 2017; Ives et al. 2018). Respondent's agreement with each statement was measured using a 5-step Likert scale with an option to skip the statement. Providing a response to each statement was compulsory.

We collected survey responses online. Initially we advertised the survey through social media using snowball sampling. To support this, we launched a press release that included information on the project and its work on August 10th, 2020. In September, we assessed the sample size and the geographical coverage of the survey in relation to population sizes of Finnish provinces. The initial sampling provided 413 responses and the data was biased towards the province of Central Finland. Therefore, we arranged an additional sampling using the services of a consulting firm, Suomen OnlineTutkimus Inc. This later sampling utilized the company's existing survey panel while excluding respondents living in Central Finland.

Appendix S2: Questionnaire form of the online survey

A Survey: My Nature Relationship

Welcome to participate in a survey examining human–nature relations in Finland! The survey is part of *Sacred Place?* research that focuses on perspectives affecting human–nature relations. Research is conducted by researchers in the University of Jyväskylä. By participating in this research, you will help us to get a more comprehensive picture of the change in perspectives.

The survey is answered anonymously and takes about 20-30 minutes to complete. Questions marked with an asterisk (*) are mandatory.

The controller of the study is the University of Jyväskylä. Before answering, please read carefully the research notification and a privacy notice containing information about the research and the processing of your personal data. The research notification and privacy notice are found here: http://www.luonnonpaikka.fi/osallistu-tutkimukseen/.

Please contact us if you have any questions.

Regards,

[Contact information masked for blind review]

* page break *

Would you kindly provide us with the following background information about yourself? They are used to compare the result with the Finnish population.

1. Home Province

- ☐ Åland
- ☐ South-Karelia
- ☐ South-Ostrobothnia
- ☐ Southern-Savonia
- ☐ Kainuu
- ☐ Kanta-Häme (Tavastia Proper)
- ☐ Central Ostrobothnia
- ☐ Central Finland
- ☐ Kymenlaakso
- ☐ Lapland
- ☐ Pirkanmaa
- ☐ Ostrobothnia
- ☐ Pohjois-Karjala
- ☐ North Osthrobothnia
- ☐ North Savo
- ☐ Päijät-Häme
- ☐ Satakunta
- ☐ Uusimaa
- ☐ South-West Finland
- ☐ Other than Finland

2. Home region *

- ☐ A densely populated area (such as a city / a village)
- ☐ A rural area (countryside)

A densely populated area is an area with at least 200 inhabitants and the distance between buildings is no more than 200 m. Areas outside of the densely populated areas are considered as rural areas.

3. Housing

- ☐ A detached house or a separate house
- ☐ A row house or a semi-detached house
- ☐ A block of a flat or a maisonette

4. Age *

- ☐ 18-24
- ☐ 25-34
- ☐ 35-44
- ☐ 45-54
- ☐ 55-64
- ☐ 65 or older

5. Gender

- ☐ Female
- ☐ Male
- ☐ Other
- ☐ I prefer not to answer

6. Highest education

- ☐ Primary school or common school
- ☐ Comprehensive school
- ☐ High school
- ☐ Vocational education
- ☐ University

7. Type of work

- ☐ Entrepreneur
- ☐ Employee
- ☐ Not employed (e.g., student, unemployed, on parental leave)
- ☐ Retired

8. I visit nature

- ☐ Every day
- ☐ 3-6 days a week
- ☐ 1-2 days a week
- ☐ Not that often

Choose 1 or 2 most important options:

9. When I visit nature, I primarily

- ☐ work
- ☐ practice my livelihood
- ☐ spend my leisure time
- ☐ exercise or otherwise maintain my physical condition
- ☐ do something else

* page break *

Choose 1 or 2 options:

10. Which of these characterizations describes your relationship with nature the best?

- ☐ Climber of heights
- ☐ Walker in peace
- ☐ Wanderer in wilderness
- ☐ Hunter or fisher
- ☐ Recreator in urban parks
- ☐ Athlete on track or in water
- ☐ Defender of nature
- ☐ Caretaker or keeper
- ☐ Watcher or observer
- ☐ Explorer or traveller
- ☐ Searcher of beauty or awe
- ☐ Nature photographer
- ☐ Sailor or boater
- ☐ Animal lover
- ☐ Green thumb or farmer

- ☐ Forest manager
- ☐ Nature professional
- ☐ Exercise practicer
- ☐ Other, what? _____________________

* page break *

Choose 1 or 2 options:

11. For me, the most important in nature is:

- ☐ Nature inside home (houseplants, pets, balcony plants...)
- ☐ Nature on my yard (the window view, garden, birds on the bird feeder…)
- ☐ Nearby nature (parks, fields, forests, water bodies, beaches, islands, cliffs, wastelands...)
- ☐ Nature at summer cottage
- ☐ Nature at my homeland
- ☐ Nature elsewhere in Finland
- ☐ Nature elsewhere than Finland
- ☐ Unique natural sites
- ☐ National landscapes
- ☐ Scenery sites
- ☐ Culturally important sites
- ☐ Historic sites
- ☐ Recreational sites
- ☐ Other, what? _____________________

12. What does ‘nature’ mean for you?

____
____
____
____

(200 characters)

13. Please write words that you consider as apt descriptions of nature.

____
____
____
____

(200 characters)

* page break *

14. What do you think about the following statements? Answer from your personal perspective. *

	Strongly disagree	Disagree	Neither agree nor disagree	Agree	Strongly agree	Doesn't apply to me
I consider the untouched nature the most impressive.						
I want to see also human handprint in the landscape.						
I know my nearby nature thoroughly.						

	Strongly disagree	Disagree	Neither agree nor disagree	Agree	Strongly agree	Doesn't apply to me
I have my own special places in nature.						
The nearby nature is better in the countryside than in the city.						
The landscapes of my birthplace are particularly beautiful.						
The landscapes of my place of residence are dear to me.						
I find that being connected to nature increases my health.						
I find that being connected to nature increases the quality of my life.						
I think nature unites Finns over the generations.						

* page break *

15. Please give us your opinion on the environmentally friendly choices in your own life? *

	Strongly disagree	Disagree	Neither agree nor disagree	Agree	Strongly agree	Doesn't apply to me
I can influence the state of nature with my lifestyle.						
I prefer to buy second-hand clothes or goods.						
I’m ashamed of flight travelling.						
I’m ready to reduce car driving for environmental reasons.						
I prefer to buy local food.						
I actively follow news about the state of nature.						
I have to change my consumption habits for nature’s benefit.						
I think ecologically sustainable living is effortless.						
I’m willing to pay more for environmentally friendly products.						
I’m ready to compensate the harm I do to nature.						

* page break *

16. What is the relationship between human and nature like, in your opinion? *

	Strongly disagree	Disagree	Neither agree nor disagree	Agree	Strongly agree	Doesn't apply to me
People have an unlimited right to utilize nature.						

	Strongly disagree	Disagree	Neither agree nor disagree	Agree	Strongly agree	Doesn't apply to me
People have the right to intervene in nature when their wellbeing or security is threatened.						
Nature can be molded to please people.						
Protecting nature today is a prerequisite of the future generations' lives.						
Nature does not need humans.						
People are more important than nature.						
Local actions create sustainability.						
People are part of nature.						
People cannot control nature.						
Nature is intrinsically valuable.						
Civic activism improves the state of nature more efficiently than legislation.						

* page break *

17. How does nature affect you? *

	Strongly disagree	Disagree	Neither agree nor disagree	Agree	Strongly agree	Doesn't apply to me
Nature calms me down.						
Nature brings me joy.						
Nature fascinates me.						
Nature invigorates me.						
I forget my worries when I'm in nature.						
I have a connection with nature.						
Nature is healing.						
Nature makes me feel small.						
The best things in nature are colors, views, or sceneries.						
The best things in nature are sounds, smells, sensations, or tastes.						

* page break *

18. Nature can cause also negative feelings. Please give us your opinion on the following statements? *

	Strongly disagree	Disagree	Neither agree nor disagree	Agree	Strongly agree	Doesn't apply to me
I don't like to go to the forest alone in the dark.						
I would not mind if all disgusting and disturbing animals and plants would disappear.						

	Strongly disagree	Disagree	Neither agree nor disagree	Agree	Strongly agree	Doesn't apply to me
Species that are harmful to people must be conserved, too.						
I don't feel comfortable in nature.						
I think environmental issues are exaggerated.						
I get anxious about news on environmental disasters.						
I feel sad because of the environmental crises caused by people.						
I feel worried because of the destruction of biodiversity.						
I feel frustrated because of the inevitability of climate change.						
I feel angry because of negligent attitudes towards nature.						

* page break *

19. How is nature present in my everyday life? *

	Strongly disagree	Disagree	Neither agree nor disagree	Agree	Strongly agree	Doesn't apply to me
I like houseplants.						
I like to potter around in the garden or in the summer cottage.						
I like to busy myself with animals.						
I want to know more about nature and understand it.						
I collect weather or nature observations regularly.						
I go into nature despite bad weather.						
Challenging terrain doesn’t bother me.						
I’m fine with being in nature, I don't need anything special to do there.						
I prefer to be alone in nature.						
I like to go for a ride with motor vehicles.						
I prefer scenic routes over highways while on the road.						

* page break *

20. What do you think about the use of the rights to roam? *

	Strongly disagree	Disagree	Neither agree nor disagree	Agree	Strongly agree	Doesn't apply to me
The best way to spend leisure time in Finland is to go out into nature.						

	Strongly disagree	Disagree	Neither agree nor disagree	Agree	Strongly agree	Doesn't apply to me
Hunting and/or fishing is important to me.						
Going out to collect mushrooms and/or berries is important to me.						
I enjoy skiing and/or cycling.						
I like to go boating and/or paddling.						
I prefer to swim in lakes, rivers, or sea.						
Camping is the best part of my nature excursions.						
Rights to roam are the cornerstone of Finns' relationship with nature.						
Rights to roam must be restricted in conservation areas.						
Finns have a closer relationship with the forest than with other habitats.						

* page break *

21. What is your opinion on protecting the following areas? *

	Strongly disagree	Disagree	Neither agree nor disagree	Agree	Strongly agree	Doesn't apply to me
Baltic Sea						
Baltic Sea coast						
Inland waters and shores						
Mires						
Forests						
Rock outcrops and scree						
Fell habitats						
Traditional rural biotopes (meadows, wood-pastures)						
Vast wilderness areas						
Rural cultural landscapes						
Green areas in cities (parks, nearby forests, and yards)						
Road verges and ruderates (e.g., roadsides, storage areas, and abandoned construction sites)						

* page break *

Thank you for your answers! Now you can close the browser.

Appendix S3: Thematic analysis on nature conceptualizations

We used two open-ended survey questions to collect qualitative data in relation to RQ1 (on ways to conceptualize nature; Fig. 2 in the article). First, the respondents defined ”nature” in their own words. Second, they listed words that they considered as apt descriptions of nature. We exported the responses and analyzed these data separately for the two questions, grouping the content of the responses under emergent themes.

In the case of nature definitions, Author4 (AML) identified repetitive topics and grouped the definitions accordingly, using MS Word. We considered each response primarily as a whole, unless it clearly included two or more topics, in which case Author4 separated the differing parts under corresponding topics. All authors discussed the initial grouping, and Author1 (KJR) developed more detailed interpretations of the definitions shared by the respondents.

As for the words associated with nature, Author1 interpreted their content in stages. In the beginning, Author1 extracted the root form for each word and counted the frequencies of the repetitive root forms using MS Excel (stage 1: root forms). This grouping revealed redundance in the form of interconnected root forms, which Author1 joined together into topics shared by the respondents (stage 2: topics). Author1 calculated the frequencies of the topics and grouped topics with similar meanings to distill the content into underlying concepts with little or no overlap (stage 3: concepts). Author1 revised the output from this analytical stage while translating the list of concepts into English. As a result, we were able to summarize the rich content of the associative words into a condensed list of nature concepts, with the frequency of each concept representing the number of related words in the original data.

Appendix S4: Factor analysis on shared nature discourses

We analyzed the quantitative data on respondents’ agreement with nature-related statements using exploratory factor analysis (to answer RQ2 on nature discourses and dimensions underlying the discourses). Here we assumed that quantifiable patterns in respondents’ statement agreement and disagreement could be translated into shared discourses.

While preparing the survey data for the factor analysis, we checked the missing data points (i.e., skipped statements: responses with “doesn’t apply to me” option selected on a statement) both according to respondents and statements. A total of 279 respondents had skipped statements using the “does not apply to me” response option.

Initially, we considered omitting the records of these 279 respondents who had skipped statements. However, that would have meant that their contribution would have been dismissed completely in the factor analysis which we considered not purposeful. Recoding, on the other hand, had the benefit of strengthening the generalizability of the results as it allowed for the larger sample size. This was important for our analysis, as we were also interested to explore the effects of the respondent background on the nature discourses.

We interpreted that those respondents, who skipped statements, had expressed inexperience or detachment to the topics of these particular statements. As we used the option “doesn’t apply to me” instead of “I don’t know”, we concluded that when choosing it, the respondents took a stance in relation whether the statement had relevance to them or not. Therefore we interpreted that the skips were, in their meaning, rather close to the non-salient middle category of “Neither agree nor disagree”. This served as the practical reasoning behind the recoding. Thus, we decided to recode these “does not apply to me” responses into the non-salient middle category in the Likert scale (“neither agree nor disagree”). In total, the recoding resulted in 721 value replacements, of which 34.8% (251) were in the nature access section, 24.1% (174) in the pro-environmental habits section, and 22.6% (163) in the nature connectedness section of the survey. Replacements in the other sections were fewer (ranging from 10 to 49). The recoding allowed us to use full data (n=726) in the factor analysis. If we had included only those respondents who provided full records, we would have lost nearly 40% of all records. We considered this loss more problematic than the recoding, which ensured broader inclusion of respondents in the factor analysis.

There were six respondents with 10 or more skipped statements (max=15). We checked the full records of these respondents to get an idea why they had provided such records. Excluding the skipped

statements, they had utilized the range of the Likert scale in the survey (i.e., not repeatedly using the non-salient middle option). We looked also at their free-text answers. One of them replied "I don't know" for questions on the character of their nature relationship, the most important things in nature, definition of nature and the words associated to nature; and another commented that she had "no relationship to forest". Three of these respondents did not provide a definition nor descriptors of nature at all. Since these respondents likely represented people who felt somewhat disconnected from nature, we thought that it was important to include them in the factor analysis, although that meant we had to recode substantial parts of their records. On the behalf of these six respondents, we recoded 74 selections (out of a total of 729; 10 % of all recodings). The other skipped statements were dispersed among the respondents. 121 respondents had skipped one statement, 51 had skipped two, 51 had skipped three, 20 had skipped four, and so forth.

Most missing data targeted certain sections and statements in the survey. Looking at which parts of the survey were most often skipped, we found out that these were related to feeling shame for flying (89 missing cases), reducing driving a car (71), or using the rights to roam: boating or canoeing (60) and camping (58). It appeared that these respondents had some limitations regarding to the activities (e.g., not owning a car). To take that into account, we derived two new dichotomic (or dummy) variables from the data to illustrate which respondents had detached themselves from the flight shame and reducing car driving; and for the utilization of rights to roam, we calculated the number of skipped statements under that section per respondent. We included these additional three variables in the factor analysis but they did not change the results.

All statistical analyses and visualizations on quantitative data were done by Author1 with computing software R version 3.6.1 (R Core Team 2019), using packages plyr (Wickham 2011), psych (Revelle 2020), and ggplot2 (Wickham 2016). We calculated a Spearman correlation matrix from the statement data and factored it using the maximum likelihood method. We used an orthogonal varimax rotation (Allen 2017) to analyze the range of discrete dimensions in the data and thus minimizing the factor-to-factor correlations. We considered that the varimax rotation would ease the interpretation of the discourses from the factors as it emphasizes differences between statement loadings. We compared factor solutions for five, six, and seven factors and chose the six-factor solution for further analysis based on scree plotting, factor eigenvalues, factor communalities, and coherence of factor interpretation.

The content of the discourses was interpreted based on statement associations within each factor and examination of statements with high loadings to each factor. We derived the standardized loadings of each statement for the resulting factors and used polarized loadings (with a value over +/- 0.4) to focus on the most informative statements in relation to each discourse. In addition, we examined statement-to-statement associations from the correlation matrix to confirm that our interpretation included all observed strong correlation patterns.

We calculated score values of the six factors for each respondent. The factor scores were used as response variables in analyzing the effect of respondent background on the discourses. In addition, we identified 20 respondents who had the highest scores for each factor. Their responses to open-ended questions were used to identify the connections between factor-based discourses (RQ2) and qualitatively interpreted nature conceptualizations (RQ1). Three respondents had high scores in two factors, and this overlap was removed by replacement to avoid pseudoreplication, so that one respondent was considered as a high scorer for one factor only (the factor with the higher score was retained).

Appendix S5: Respondents' background

We made statistical summaries on the respondent background variables to gain better understanding on the sample of our survey. We also wanted to derive information on possible impacts of the respondents' background on the discourses. We examined the effect of selected background variables on the factor scores using generalized linear modelling (GLM) with Gaussian error distribution and a log link function. We chose Gaussian distribution as our response variables (i.e., the factor scores) were continuous and included both negative and positive values. The variable distributions were not normal but left-skewed, and therefore we used the log link function that is better suited for skewed data. The log link requires that values of the response variable are positive, and treats the relationship between the response variable and its regressors as multiplicative (Equations 1 & 2):

$$log_e(\mu_i + k) = \alpha + \beta_1 x_{i1} + \beta_2 x_{i2}$$ [Eq. 1]

or:

$$\mu_i = \exp(\alpha) * \exp(\beta_1 x_{i1}) * \exp(\beta_2 x_{i2}) - k$$ [Eq. 2]

where:

μ = expected factor score

α = intercept

β = coefficient(s)

x = regressor(s)

k = a constant to transform the factor score ranges into positive values (here, 6 was used)

To avoid collinearity among the regressors in the GLMs, we examined the possible dependencies of the background variables using pairwise non-parametric statistical tests. We chose the suitable test based on response scale (categorical or ordinal, depending on the variable). In the below table, significance levels under $p = 0.001$ are expressed with $p < 0.001$. Statistically significant test results ($p \leq 0.05$) indicate that the two variables are dependent from each other and thus cannot be used as separate regressors in a generalized linear model.

**Variable 1**	**Variable 2**	**Test**	**p-value**	**Dependence**
Gender	Level of education	Khii square	0.024	yes
Gender	Living environment	Khii square	0.062	no
Gender	Age	Kruskal-Wallis	< 0.001	yes
Gender	Frequency of nature visits	Kruskal-Wallis	0.0234	yes
Frequency of nature visits	Living environment	Mann-Whitney U	< 0.001	yes
Frequency of nature visits	Age	Spearman rank correlation	0.824	no
Frequency of nature visits	Level of education	Kruskal-Wallis	0.452	no
Level of education	Age	Kruskal-Wallis	< 0.001	yes

As a result, we excluded interdependent background variables (home region, gender, and education) from the GLMs. We included respondent age and frequency of going into nature as regressors for the factor scores in the GLM analyses. These two variables were independent of each other.

To ease the execution and interpretation of the GLMs, we transformed the ordinal scale regressors into interval scale by using the mid-points of the ordinal classes as input data (Williams 2020). Respondent age had six classes and we recoded the classes using the mid-age of each class as the new value. An exception was made with the last age class with the oldest respondents, categorized as ”65 years or older”. Here we used the average of the life expectancy of Finnish females (85 years) and males (79 years) to calculate the class maximum, weighted according to the observed gender distribution within the class (61 female and 41 male). The calculations resulted in the maximum of 83 years and the mid-age of 74 years for the last age class. The frequency of going into nature was an ordinal variable with four classes, which indicated how often the respondent visited nature on a weekly basis. Here the transformation into interval scale was done by dividing the number of weekly nature visits with the number of weekdays, i.e. for class ”Everyday”: 7/7=1. For middle range classes, class mid-points were used in the calculation. For class ‘Not that often’ a value of 0.5 was used to indicate that these respondents did not go into nature every week but could do that in every other week (0.5/7=0.0714).

Appendix S6: Audience interviews

To enrich our inquiry into human–nature relations by incorporating experiential aspects, Author4 organized a site-specific walking performance on a conservation area and Author2 (AKT) and Author3 (RN) conducted audience interviews after the performance (Fig. 2 in the article). The performance took place five times a day during 22nd–23rd of August, 2020. It included a guided walk trespassing the Hitonhauta conservation area in Central Finland, near the border of Laukaa and Äänekoski municipalities. Eight acts were performed at different locations along the walking route. Audience stopped to view these short acts by performers that represented different historical aspects of human–nature relations in Finland. The transitions in-between the acts were walked in silence. During the transitions, the members of the audience could observe the surroundings and connect with nature and each other non-verbally. A detailed description of the arts-based research approach is published separately (Niemelä et al. 2023).

After the performance, the audience could voluntarily participate in a structured research interview. We assumed that attending the performance would motivate and encourage participants to reflect on their ideas of nature and explain their nature relationship, easing them to verbalize their experience of nature. At the onset of the interview, participants gave their informed consent and agreed with data collecting privacy policy and audio-recording. As the event was public, we did not collect participants' background information to ensure their anonymity. Participants were interviewed individually or in pairs. Each interview lasted approximately 5–10 minutes.

A total of 140 persons attended the site-specific walking performance, while the maximum amount of audience permitted by the environmental authorities was 150. From the full audience, 71 persons participated in the research interview to reflect on their experience.

The interview consisted of a following set of questions:

1. Was Hitonhauta gorge already familiar to you?

    [if yes:] Did the performance give some new viewpoint to the gorge area?

    [if no:] What was your first encounter with the Hitonhauta gorge like?

2. In your opinion, what was nature's role like in the performance?

3. Was there something in the walking performance you felt particularly close to your own relation with nature?

During the winter 2020–2021, the interviews were transcribed verbatim and their content was qualitatively analyzed by Author2 and Author3. The content analysis included deductive coding of the transcripts (Elo and Kyngäs 2008), using Atlas.ti program. Author2, Author3, and Author1 developed the code system (Appendix S7; Table 5 in the article) in between the transcription and the coding stages. The codes and code groups were informed by RQ3 (What kinds of embodied and emotional nature experiences emerged from participating in a site-specific walking performance?). The aim of the deductive coding was to detect which parts of the data were most informative according to RQ3.

After the deductive coding was finished, Author2 and Author1 used Atlas.ti's query tool to search for overlapping and neighboring co-occurrences of code groups Emotions, Actions, Place, and Walking performance (Fig. 3). The derived parts of the data were coded again by Author2, but this time we analyzed the content inductively according to emergent themes (Elo and Kyngäs 2008).

Appendix S7: Code system for the deductive content analysis

Code group: **Walking performance**: topics related to the artwork, performance, performing

Codes:

- [**Performance in general**] mentions or accounts discussing the walking performance in general and when talking about the whole performance / artwork.
- [**Uniqueness**] accounts discussing a single time of performance; the performance ran for ten times.

- [**From spirits of forest to forest industry**] first act in the walking performance: “The tree spirits and the development of technology” (1st scene). Societal changes during the 20th century, technology, growth, wellbeing, development, from agrarian society to urban society.
- [**Logger of the trees**] first act in the walking performance: “The tree spirits and the development of technology” (2nd scene). Enjoyment of nature work: the logger.
- [**Historical eras**] second act in the walking performance: “Rocks, ice, human”. Earth 4000 million years ago, the extinction of the dinosaurs 65 million years ago, the appearance of human species 2,5 million years ago, the last Ice Age 11 000 years ago, human-caused climate change, melting of continental ice sheets.
- [**Mother Nature**] third act in the walking performance: “Mother Nature, seeking nature connectedness". The aspiration to be united with nature, spirituality, nature as a healing force, energy.
- [**Antagonistic Nature**] fourth act in the walking performance: “Nature as a fierce opponent” (hymn When Does the Morning Star Light Up by Anders Odhelius, 1745). Struggle for survival, suffering, the great famine years of the 19th century, crafting skills, searching of nutrition and materials from nature, Christianity, surviving, hunger, fatigue.
- [**Cycles of nature**] fifth act in the walking performance: “The cycle of nature, fast and slow cycles”. Changes in nature, the human body as consisting of elements and returning back to elements, chemical elements and compounds, chemical processes, the movement of the Earth crust.
- [**Mythic nature**] sixth act in the walking performance: “Grove of Tuoni, grove of night” (composition by Jean Sibelius for the poem The Song of my Heart by Aleksis Kivi, 1870). National romanticism, nature in art, the meanings of nature, Kalevala (the national epic of Finland), illuminated vocabulary, death.
- [**Conservation of nature**] seventh act in the walking performance: “Nature conservation” (monologue based on writings of Pentti Linkola (1932–2020)). The rise of conservationism, deep ecology, choices of the individual, consequences of our deeds, loneliness, the acts of human communities, defence mechanisms, humanity, humanism, climate anxiety, ecoanxiety.
- [**Transitions**] mentions or accounts discussing transitions between the acts, walking.

Code group: **Place**: topics related to place and site

Codes:

- [**Hitonhauta gorge**] accounts related to the site of the performance, the Hitonhauta conservation area and its surroundings (as a natural site or as a venue of performance).
- [**Nature as a stage**] reflections on the stages of the acts, theater, or nature as a stage of the performance.
- [**Sense of place**] place experience, meanings given to the place, characterization of the place, emotional connection to the place.
- [**Weather, seasons**] observations on weather or seasons.
- [**Landscape**] mentions of views, sceneries, or other (visual) observations related to aesthetics.
- [**Biotic nature**] observations on biotic elements of nature (trees, animals, etc.).
- [**Abiotic nature**] observations on abiotic elements of nature (rocks, water, etc.).
- [**Habitat types**] observations on habitats (forest, mire, boulder field, etc.)
- [**Path**] observations on/of the path, the terrain, or the route.
- [**Space**] mentions related to Euclidian concept of space; space as physical, homogeneous environment.

Code group: **Views and conceptions**: topics related to conception, notion, perception, view

Codes:

- [**Cultural traditions**] Finns’ relations with nature, closeness of nature in everyday life in Finland, cultural understandings.
- [**Health effects**] reflections on empowerment, healing, or other health effects of nature.

- [**Nature as a retreat**] reflections on nature as a retreat, a refuge, or a sanctuary.
- [**Mythology**] reflections on Finnish folklore, often related to forest, and animistic mythology; (e.g., forest spirits, experience of ‘forest cover’ as a transcendental place, or mentions of Mother Earth or Gaia.
- [**Nature conservation**] reflections on nature conservation and protection, politics, policies, practices.
- [**Utilitarianism**] accounts related to utilitarianism (including both material benefits such as income and immaterial benefits such as health effects).
- [**Religiousness**] Mentions of religiosity, religions, or other religionist reflections.
- [**Value choices and value diversity**] Reflections on nature-related value choices and value diversity.
- [**Nostalgy**] accounts regarding the nostalgy for nature, longing for natural past, nature-related memories and experiences in one’s own life.

Code group: **Emotions**: topics related to emotions, feelings, affectivity, experience

Codes:

- [**Memories**] participants’ reflections on their lives and experiences, memories triggered by the performance.
- [**Sacredness, spirituality**] accounts related to spirituality, including nature mysticism.
- [**Emotion**] accounts regarding feelings and emotions (positive, negative, or ambiguous) or otherwise indicating emotional experience or reactions.
- [**Embodied experiences**] observations on and accounts regarding or indicating bodily experience, corporeality, or embodied activities.
- [**Sensory experience**] observations on and accounts regarding or indicating sensations, multisensory experience, sensory stimuli.
- [**Experiencing awe in nature**] observations on and accounts regarding or indicating experience of astonishment in nature, overwhelm, or being at the mercy of nature, valuing the wilderness or the untouched nature.
- [**Being impressed by the performance**] accounts regarding or indicating being impressed or affected by the performance; responsiveness to the performance or its parts, mental and physical reactions.
- [**Experiencing a sense of fracture**] accounts regarding or indicating an awakening experience, experience of becoming aware of something.
- [**Interpretations of the performance**] interpretations given to the performance or its acts.
- [**Bonding with nature**] accounts regarding or indicating a sense of nature connection, or experience of merging with nature.

Code group: **Actions**: topics related to actions, behaviors, practices

Codes:

- [**Being in silence**] silence (in nature / of the performance), being silent.
- [**Staying still**] stopping, staying still, slowing down.
- [**Customs and habits linked to nature relation**] social habits, customs and behaviors, and deviations from them.
- [**Group and group dynamics of a certain act**] sense of connection with the group, an experience shared with others, a sense of community (experience).
- [**Perceiving**] active observations and sensuous experiences.
- [**Rights to roam**] reflections on accessing nature (using the rights to roam, a.k.a. the everyman's rights), hobbies in nature.
- [**Feeling harmony**] accounts on the sense of balance or harmony (of nature / in the performance / of oneself).
- [**Livelihood as a mode of utilitarianism**] accounts regarding livelihood, income, business, work.

- [**Pace of the walking**] observations on the rhythm, pace, speed (e.g., the group's walking pace, rhythms of nature)
- [**Nature contradiction recognized**] reflections on the contradictions between one's own actions and conception of nature.

Code group: **Human–nature**: topics related to human–nature interactions, positions between people and nature

Codes:

- [**Humans as part of nature**] accounts positioning humans as part of nature.
- [**Generations**] reflections regarding human–nature relations over the generations, or generational differences / similarities.
- [**Humans as separate from nature**] accounts positioning humans as separate from nature.
- [**Feeling small in face of nature**] accounts positioning humans at the mercy of nature.
- [**Agency of nature**] accounts or observations of the agency of nature, personifications of nature, or nature as the protagonist (or antagonist) in the performance.
- [**COVID-19**] reflections on the effects of COVID-19 pandemic.
- [**Respect towards nature**] reflections on the respect towards nature.
- [**Geological time**] reflections on geological history (incl. glacial periods), or the time span exceeding that of humans, perceptions of time, observations of the signs of the Ice Age.

References

Allen, M. 2017. Factor Analysis: Varimax Rotation. In *The SAGE Encyclopedia of Communication Research Methods*, 531–534. Thousand Oaks, California,USA: SAGE Publications, Inc. doi:10.4135/9781483381411.n191.

Braito, M. T., K. Böck, C. Flint, A. Muhar, S. Muhar, and M. Penker. 2017. Human-Nature Relationships and Linkages to Environmental Behaviour. *Environmental Values* 26: 365–389. doi:10.3197/096327117X14913285800706.

Cronon, W. 1996. The trouble with wilderness: or, getting back to the wrong nature. *Environmental History* 1: 7–28.

Elo, S., and H. Kyngäs. 2008. The qualitative content analysis process. *Journal of Advanced Nursing* 62: 107–115. doi:10.1111/j.1365-2648.2007.04569.x.

Flint, C. G., I. Kunze, A. Muhar, Y. Yoshida, and M. Penker. 2013. Exploring empirical typologies of human–nature relationships and linkages to the ecosystem services concept. *Landscape and Urban Planning* 120. Elsevier B.V.: 208–217. doi:10.1016/j.landurbplan.2013.09.002.

Haila, Y. 1999. Socioecologies. *Ecography*. doi:10.1111/j.1600-0587.1999.tb00571.x.

Ives, C. D., and J. Fischer. 2017. The self-sabotage of conservation: reply to Manfredo et al. *Conservation Biology* 31: 1483–1485. doi:10.1111/cobi.13025.

Ives, C. D., M. Giusti, J. Fischer, D. J. Abson, K. Klaniecki, C. Dorninger, J. Laudan, S. Barthel, et al. 2017. Human–nature connection: a multidisciplinary review. *Current Opinion in Environmental Sustainability* 26–27: 106–113. doi:10.1016/j.cosust.2017.05.005.

Ives, C. D., D. J. Abson, H. von Wehrden, C. Dorninger, K. Klaniecki, and J. Fischer. 2018. Reconnecting with nature for sustainability. *Sustainability Science* 13: 1389–1397. doi:10.1007/s11625-018-0542-9.

Kellert, S. R. 2006. Building for life: Designing and understanding the human-nature connection. *Renewable Resources Journal* 24.

Kontula, T., and A. Raunio. 2018. *Threatened habitat types in Finland 2018. Red List of habitats, part I: Results and basis for assessment (In Finnish with English summary)*. Edited by Tytti Kontula and Anne Raunio. Helsinki, Finland: Finnish Environment Institute and Ministry of the Environment.

Lumber, R., M. Richardson, and D. Sheffield. 2017. Beyond knowing nature: Contact, emotion, compassion, meaning, and beauty are pathways to nature connection. Edited by Brock Bastian. *PLOS ONE* 12: e0177186. doi:10.1371/journal.pone.0177186.

Manfredo, M. J., J. T. Bruskotter, T. L. Teel, D. Fulton, S. H. Schwartz, R. Arlinghaus, S. Oishi, A. K. Uskul, et al. 2017. Why social values cannot be changed for the sake of conservation. *Conservation Biology* 31: 772–780. doi:10.1111/cobi.12855.

Muradian, R., and U. Pascual. 2018. A typology of elementary forms of human-nature relations: a contribution to the valuation debate. *Current Opinion in Environmental Sustainability* 35. Elsevier B.V.: 8–14. doi:10.1016/j.cosust.2018.10.014.

Niemelä, R., A.-M. Laulumaa, A.-K. Tupala, and K. J. Raatikainen. 2023. A detour in research through the gorge: Approaching human–nature connections with site-specific performance. *Applied Theatre Research* 11. Intellect: 71–92. doi:10.1386/atr_00077_1.

R Core Team. 2019. *R: A language and environment for statistical computing*. Vienna, Austria: R Foundation for Statistical Computing.

Revelle, W. 2020. *psych: Procedures for Psychological, Psychometric, and Personality Research*. Northwestern University, Evanston, Illinois, USA.

Sandbrook, C., J. A. Fisher, G. Holmes, R. Luque-Lora, and A. Keane. 2019. The global conservation movement is diverse but not divided. *Nature Sustainability* 2. Nature Publishing Group: 316–323. doi:10.1038/s41893-019-0267-5.

Wickham, H. 2011. The Split-Apply-Combine Strategy for Data Analysis. *Journal of Statistical Software* 40: 1–29. doi:10.18637/jss.v040.i01.

Wickham, H. 2016. *ggplot2: Elegant Graphics for Data Analysis*. Springer-Verlag, New York, USA.

Williams, R. 1980. Ideas of Nature. In *Problems in Materialism and Culture: Selected Essays*, 67–85. London, United Kingdom: Verso.

Williams, R. A. 2020. Ordinal Independent Variables. In *SAGE Research Methods Foundations*, ed. P. Atkinson, S. Delamont, A. Cernat, J. W. Sakshaug, and R. A. Williams. London, United Kingdom: SAGE Publications Ltd. doi:10.4135/9781526421036938055.
